# In vivo evaluation of a Nano-enabled therapeutic vitreous substitute for the precise delivery of triamcinolone to the posterior segment of the eye

**DOI:** 10.1007/s13346-024-01566-1

**Published:** 2024-03-22

**Authors:** Kruti Naik, Lisa Claire du Toit, Naseer Ally, Yahya Essop Choonara

**Affiliations:** 1 Wits Advanced Drug Delivery Platform Research Unit, Department of Pharmacy and Pharmacology, School of Therapeutic Sciences, Faculty of Health Sciences, University of the Witwatersrand, 7 York Road, Johannesburg, Parktown, 2193 South Africa; 2 Division of Ophthalmology, Department of Neurosciences, School of Clinical Medicine, Faculty of Health Sciences, University of the Witwatersrand, 7 York Road, Johannesburg, Parktown, 2193 South Africa

**Keywords:** Vitreous substitute, *In situ* hydrogel, Polymers, Retinal detachment, Nanoparticles, Triamcinolone acetonide

## Abstract

This study focused on the design of a thermoresponsive, nano-enabled vitreous substitute for the treatment of retinal diseases. Synthesis of a hydrogel composed of hyaluronic acid and a poloxamer blend was undertaken. Poly(D,L-lactide-co-glycolide) acid nanoparticles encapsulating triamcinolone acetonide (TA) were synthesised with a spherical morphology and mean diameter of ~ 153 nm. Hydrogel fabrication and nanoparticle loading within the hydrogel was confirmed via physicochemical analysis. Gelation studies indicated that hydrogels formed in nine minutes and 10 min for the unloaded and nanoparticle-loaded hydrogels, respectively. The hydrogels displayed in situ gel formation properties, and rheometric viscoelastic studies indicated the unloaded and loaded hydrogels to have modulus values similar to those of the natural vitreous at 37 °C. Administration of the hydrogels was possible via 26G needles allowing for clinical application and drug release of triamcinolone acetonide from the nanoparticle-loaded hydrogel, which provided sustained in vitro drug release over nine weeks. The hydrogels displayed minimal swelling, reaching equilibrium swelling within 12 h for the unloaded hydrogel, and eight hours for the nanoparticle-loaded hydrogel. Biodegradation in simulated vitreous humour with lysozyme showed < 20% degradation within nine weeks. Biocompatibility of both unloaded and loaded hydrogels was shown with mouse fibroblast and human retinal pigment epithelium cell lines. Lastly, a pilot in vivo study in a New Zealand White rabbit model displayed minimal toxicity with precise, localised drug release behaviour, and ocular TA levels maintained within the therapeutic window for the 28-day investigation period, which supports the potential applicability of the unloaded and nanoparticle-loaded hydrogels as vitreous substitutes that function as drug delivery systems following vitrectomy surgery.

**Graphical Abstract:**

**Fig. a:**
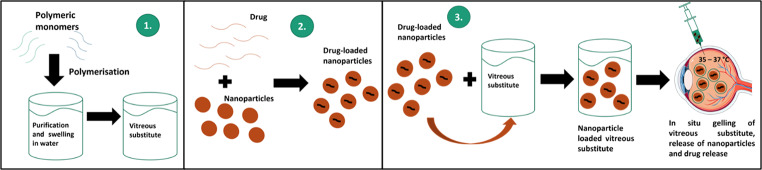
Schematic representation of the nano-enabled therapeutic vitreous substitute synthesis process. This figure was partly generated using Servier Medical Art, provided by Servier, licensed under a Creative Commons Attribution 3.0 unported license

**Supplementary Information:**

The online version contains supplementary material available at 10.1007/s13346-024-01566-1.

## Introduction

The vitreous is a clear, gel substance that occupies the majority of the posterior segment of the eye [1, 2]. It is considered to be an avascular and hydrated extracellular gel matrix [3, 4]. This gel matrix is composed mainly of water, collagen, and hyaluronic acid (HA) constituting a volume of 4 mL [5, 6]. Type II collagen, a fibrous extracellular matrix (ECM) protein, has a rigid structure that forms a network with HA in the vitreous [7, 8]. HA is a glycosaminoglycan (GAG) that stabilises the collagen, creating an osmotic pressure that creates the tamponade effect holding the retina in place [9, 10]. This network formed by collagen and HA is responsible for the behaviour of the vitreous as a viscoelastic solid allowing for the absorption of shocks and vibrations experienced by the eye, thereby protecting the surrounding intraocular tissues [11, 12].

The current treatment for various vitreoretinal diseases is vitrectomy, whereby the damaged vitreous is removed and replaced by silicone oil [13, 14]. Other substitutes such as perfluorocarbon liquids and air are also used, however, silicone oil is the first choice for substitution [2, 15]. Adverse effects associated with silicone oil substitution such as cataracts and cytotoxicity have resulted in a need for a substitute that can mimic the natural vitreous [16, 17].

Vitreous substitution with in situ-forming hydrogels for retinal stabilisation following vitrectomy has been extensively studied. These hydrogels have been studied with natural polymers, such as HA [18–20], gellan gum [21] and chitosan [22, 23]; and synthetic polymers such as methylcellulose [24, 25], polyacrylamide [26, 27], polyvinyl alcohol [28–30], and polyethylene glycol [31–33]. Advantages of in situ-forming hydrogels include the ability to tune the mechanical and optical characteristics to mimic those of the natural vitreous, and the easy injectability of the substitute using current vitrectomy procedures.

The in situ hydrogels for vitreous substitution studied to date have predominantly focused on native vitreous mimicry. They have mimicked the mechanical and optical properties of the natural vitreous. A study by Tram et al. [33] further investigated a PEG-based hydrogel that releases an antioxidant. However, these studies have not considered the treatment of any underlying posterior segment ocular diseases, or the inflammation (endophthalmitis) caused by pars plana vitrectomy [34, 35] that was observed as a result of those studies.

Intravitreal drug treatment is the most direct method of drug delivery to treat posterior segment diseases. Despite advantages such as increased drug concentration and decreased systemic effects [36, 37], intravitreal injections have to be administered frequently to maintain the therapeutic drug window [38, 39]. This results in a higher risk of ocular inflammation and cataracts. Therefore, long-term drug delivery using nano-systems and hydrogel systems is being investigated. However, previous studies based on the intravitreal delivery of drugs using hydrogels have focused on systems that combine with the natural vitreous and are not vitreous substitutes [40–42]. More recently, several advances have been underway for application of the vitreous substitute as both a tamponade as well as a drug release system for the treatment of ocular diseases [43]. The requirement for multiple intravitreal injections could be reduced or eliminated by employing a suitable vitreous substitute that delivers the drug over an extended period, thus promoting patient compliance and acceptance. Schulz and Szurman [43] delineated the requirements for vitreous substitutes, defining both the current clinical standard in addition to novel polymer-based vitreous substitutes as drug delivery systems in terms of their release mechanisms, efficiencies, challenges, and future potential. Recently, polymer-based vitreous substitutes have been investigated as drug delivery systems for the delivery of various bioactives, including anti-inflammatory agents [44], cytostatics, antioxidants [33, 45] and proteins [46, 47]. While approaches based on current clinically used endotamponades (balanced salt solution and silicone oil) have already been investigated in humans, these novel hydrogel-based vitreous substitutes have only been evaluated in preclinical studies in vitro and in rabbits and have not attained clinical translation. Schulz and Szurman [43] highlighted that the paradigm shift from hydrophobic to hydrophilic vitreous substitutes and the associated focused research effort advocates clinical translation of hydrogel-based vitreous substitutes within the next few years. Clinically relevant release over a few weeks would be a requirement for the reduced the recurrence rate of ocular diseases such as proliferative vitreoretinopathy (PVR), which is a severe complication of vitreoretinal surgery due to a complex cellular reaction, where vitreoretinal wound healing occurs, resulting in tractional retinal detachment [45]. Following the global surgical standard treatment of vitrectomy, a tamponade agent in the form of a vitreous substitute is necessitated to occupy the vitreous cavity and aid reattachment of retina for a reduction in the recurrence of postoperative retinal detachment. Exploiting this substitute as a drug delivery system could further enhance the postoperative treatment and prognosis of PVR [45].

Ultimately, the approach of applying vitreous substitutes as drug delivery systems would gain from adapting the various existing hydrogel-based delivery systems, which are primarily delivered to the vitreous cavity by intravitreal injection, to the requirements of vitreous substitutes. Further considerations include ensuring that the incorporated drug does not affect optical properties (transparency and refractive index) and thus patient vision, aligning the mechanical properties/viscoelasticities of the drug release system to the native characteristics of the healthy human vitreous to avoid mechanical damage to surrounding tissue structures, adjusting the degradation/release profile to align with the treatment periods of the specific ocular diseases, as well as consideration of the incorporation of degradable nanoparticles or microparticles to promote the sustained release of drugs from polymer-based vitreous substitutes [43]. Donati et al. [5] elaborated on an ideal testing protocol for evaluation of the optimal vitreous substitute which includes determination of light transmittance, hydration and water swelling, oscillatory and shear-stress analysis, shear-creep analysis, in vitro and in vivo biocompatibility, and degradation assessment.

HA, a major component of the natural vitreous, is a linear polysaccharide that is composed of repeating disaccharide units (D-glucaronic acid and N-acetyl-D-glucosamine) that are linked via β-glycosidic bonds [48, 49]. As it is an endogenous substance in the eye, HA and HA-based hydrogels have been widely studied for ocular administrations [50–52]. HA-based hydrogels for vitreous substitution have been studied using non-modified and modified HA. The integration of HA into hydrogel systems via physical mixing can avoid cytotoxic effects that may occur due to crosslinkers in modified-HA hydrogels [49, 53]. The mixing of HA with synthetic polymers that exhibit thermo-responsive properties can allow for HA-based in situ-forming hydrogels.

Poloxamers are synthetic triblock copolymers composed of polyethylene oxide (PEO) and polypropylene oxide (PPO) [54, 55]. Poloxamers are available in a variety of molecular weights that modify their gelation behaviour [56, 57]. Poloxamers undergo sol-gel phase transitions due to the formation of micelles as a result of the organisation of monomers into ordered structures as the temperature of the poloxamer solution increases [58, 59]. Poloxamers display a reversible thermo-responsive behaviour, whereby a sol-gel phase transition occurs around physiological temperature (~ 37 °C) and a reverse gel-sol phase transition occurs around 50 °C [57, 60].

Vitreous substitutes composed of poloxamers have been investigated. These studies were conducted only with P407 and required large concentrations of P407 resulting in the occurrence of ocular toxicity [61–63]. Poloxamer-based hydrogels for ophthalmic administration with a combination of P407 and P188 were formulated and found to be non-toxic and non-irritating [64, 65]. This is likely because the addition of P188 required a lower concentration of P407 reducing the ocular toxicity effects noted with P407 in isolation [66–68].

Polymeric nanoparticles have been employed for drug delivery for a considerable time as they are relatively easy to prepare, they can efficiently incorporate a variety of drugs and bioactive compounds within them and provide a controlled release of those substances [69, 70]. Poly(DL-lactic-co-glycolic) acid (PLGA) is a biodegradable and biocompatible, FDA-approved polymer that has displayed great potential in drug delivery [71, 72] and it has been used to deliver many ocular therapeutic agents [73–76]. PLGA nanoparticles can control and extend the release of drugs and biomolecules in the eye, however, intravitreal nanoparticles are free-moving within the vitreous resulting in an increased clearance of the drugs [39, 77, 78]. Encapsulation of nanoparticles within hydrogels can provide them with a stable platform for more precise delivery within the vitreous. This double-drug delivery system can also reduce the initial burst release of drugs normally observed with nanoparticle drug delivery [79–81].

Therefore, the objective of this study was to fabricate a hydrogel with a dual purpose for the treatment of posterior segment diseases of the eye, such as PVR. The first is to function as a vitreous substitute/ tamponade and the second is ocular nanomedicine for postoperative management of inflammation following vitrectomy and/or subsequent treatment of the vitreoretinal disorder by acting as a drug reservoir with drug-loaded nanoparticles resulting in sustained and more precise drug release within the posterior segment, thereby eliminating the need for frequent injections. This study, therefore, sought to formulate an in situ-forming hydrogel composed of the natural polymer, HA, and a blend of different molecular weight poloxamers (P188 and P407). Drug-loaded PLGA nanoparticles were subsequently encapsulated in the formulated hydrogel. Triamcinolone acetonide, a corticosteroid drug used in the treatment and management of vitreoretinal diseases such as PVR, uveitis, and macular degeneration, was used for this study as a model anti-inflammatory hydrophobic drug [82–84] for demonstration of its encapsulation and extended release from a vitreous substitute compared to previous investigations [44]. Thereafter, the nanoparticles and the hydrogel (unloaded and nanoparticle-loaded) were characterised and assessed for viscoelastic properties, optical properties, nanoparticle size and morphology, drug release characteristics, and biocompatibility. Lastly, a pilot in vivo biocompatibility and drug release study of the nanoparticle-loaded hydrogel was conducted over 28 days in New Zealand Albino rabbits.

## Materials and methods

### Materials

The materials used in this research were bought from Sigma-Aldrich (St Louis, MO, USA). These materials include Poly(D,L-lactide-co-glycolide) (PLGA) (LA/GA = 50/50, MW = 45 000), Poly(vinyl alcohol) (PVA) (MW = 85 000-124 000, 99+% hydrolysed), Dichloromethane, Ethyl Acetate, Triamcinolone acetonide, Poloxamer 407 (Pluronic ® F-127), Poloxamer 188 (Pluronic ® F-68), Hyaluronic acid sodium salt (from *Streptococcus equi*), and Lysozyme (from chicken egg white) (~ 70,000 U/mg).

### Synthesis of the poloxamer and hyaluronic acid hydrogel

The thermo-responsive hydrogel was formulated according to the cold method. Briefly, known concentrations of Poloxamer 188, Poloxamer 407 and Hyaluronic acid were mixed in deionised water on magnetic stirring at 4 °C for 12 h. The solution was kept at 4 °C overnight for complete homogenisation.

The solution was then equilibrated before visual inspection of gelation, after which a vial inversion test was performed. For the inversion test [85], 4 mL of the prepared formulation was placed in a 10 mL glass vial. It was then immersed in a water bath with a temperature of 37 °C. The time at which no flow of the sample was observed was considered to be the formation of the gel and the sol-gel phase change was visualised by the inversion of the vial. The temperature of gelation was also measured. The gelation temperature was confirmed as the temperature at which the gel remained intact after the vial was inverted for 60 s.

### Preparation of PLGA nanoparticles and loading of nanoparticles in the hydrogel

Triamcinolone acetonide was encapsulated into PLGA nanoparticles using a double-emulsion solvent evaporation method. Various formulations were prepared and screened to determine the correct organic mixture ratio for nanoparticle synthesis. Triamcinolone acetonide of known mass was added to 2 mL of an organic mixture of ethyl acetate and dichloromethane (various ratios of 1:1, 1:2, 1:3, 2:1, and 3:1) containing 10 mg PLGA (slow stirring). This prepared solution was transferred, dropwise with a 26-gauge needle, into a 20 mL PVA solution (1% ^w^/_v_) and stirred for 5 min. The solution was sonicated in an ice bath for 6, 20-second cycles (130 W, 20 kHz, 50% Amplitude) with a probe sonicator (Sonics, Ultrasonic Processor, VCX130, Newtown, CT, USA). Thereafter, the solution was stirred for 48 h for evaporation and the hardening of the triamcinolone acetonide-loaded nanoparticles. The nanoparticles were collected via centrifugation and washed with deionised water after which they were lyophilised (Labconco FreeZone 12 Console Freeze Dry, Kansas City, USA).

For nanoparticle loading within the hydrogel, the optimised nanoparticles were prepared as above. Following lyophilisation, nanoparticles were resuspended in deionised water. The resuspended nanoparticle solution was gently sonicated for 2, 20-second cycles (130 W, 20 kHz, 10% Amplitude) to agitate and separate nanoparticle aggregates allowing for an optically transparent nanoparticle solution. The polymers for the hydrogel were mixed in the resuspended nanoparticle solution rather than deionised water, as described above in Section “Synthesis of the poloxamer and hyaluronic acid hydroge”.

### Nanoparticle characterisation and quantification of nanoparticle loading in hydrogel

Triamcinolone acetonide-loaded nanoparticles and unloaded nanoparticles were screened based on their diameter size, particle dispersity, surface charge, and encapsulation and loading of the drug. The Zetasizer NanoZS (Malvern Instruments Ltd., Worcestershire, UK) was used to determine the nanoparticle size, polydispersity index (PDI) and surface charge. The PDI was used as an indicator of dispersity where values ≤ 0.5 indicate monodisperse particles and ≥ 0.5 indicated polydisperse particles. The nanoparticle formulations were resuspended in deionised water (1 mg of the formulation in 1 mL water) and analysed using dynamic light scattering (DLS). The sample temperature was maintained at 25 °C and samples were diluted and sonicated prior to the measurement. The nanoparticle formulations were screened based on their size, PDI and surface charge. Formulations with favourable results, namely particle size < 200 nm, PDI ≤ 0.2, and a negative surface charge, were then screened based on the encapsulation efficacy and drug loading capacity. All measurements were conducted in triplicate.

The encapsulation efficiency of the nanoparticles was calculated using Eq. 1.

1 $$ Encapsulation \,efficiency \left(\text{\%}\right)=\frac{{W}_{L}}{{W}_{O}}\times 100$$

Where W_0_ is the initial mass of triamcinolone acetonide and W_L_ is the mass of triamcinolone acetonide loaded in the nanoparticles. Encapsulation efficiency is the percentage of drug that is entrapped in the nanoparticle with respect to the amount of drug that was used in the nanoparticle preparation. Quantification of encapsulation efficiency via determination of the W_L_ was performed using a UV/Vis spectrophotometer (Lambda 25, PerkinElmer, MA, USA).

The surface morphology of the formulation selected (based on the above parameters) was then determined. Lyophilised samples of the drug-loaded, and unloaded nanoparticles were redispersed in deionised water and one drop was placed on an aluminium stub and allowed to dry for 24 h. Samples were coated in a vacuum with one layer of gold using a sputter coater to induce induction. A field emission scanning electron microscope (Zeiss, Jena, Germany) under an argon atmosphere was used to analyse the morphology of each sample.

Lyophilised samples of the drug-loaded nanoparticles were weighed prior to resuspension in deionised water (4 mL) which subsequently underwent hydrogel formation as discussed in the Section “Synthesis of the poloxamer and hyaluronic acid hydroge”. This allowed for the complete embedding of the nanoparticles within the hydrogel for administration. The amount of drug in the final hydrogel is therefore the amount of drug quantified to be encapsulated with a specific mass of nanoparticles as per Eq. 1.

### Physicochemical characteristics of the nanoparticles and the hydrogel

#### Molecular transition and interaction analysis

Fourier Transform Infra-Red (FTIR) analysis was conducted on all pure polymers, lyophilised unloaded and loaded nanoparticles, and unloaded and loaded hydrogels. The molecular transitions and interactions were identified using a PerkinElmer ATR-FTIR (Spectrum 2000, Waltham, MA, USA) fitted with a single reflection diamond MIRTGS detector. FTIR spectra (20 scans per spectrum) were obtained using a wavelength range of 4000 − 650 cm^− 1^, a resolution of 4 cm^− 1^, and a constant pressure of 110 psi.

#### Thermal transitions and properties analysis

The thermal properties of the pure polymers, the lyophilised unloaded and loaded nanoparticles, and the lyophilised unloaded and loaded hydrogels were determined using a Differential Scanning Calorimeter (DSC) (Mettler Toledo DSC, STARe system, Schwerzenback, Switzerland) and a Thermogravimetric analyser (TGA) (PerkinElmer, TGA 4000, Llantrisant, Wales, UK). Thermally-induced phase transitions of the samples were determined using the DSC in an inert N_2_ atmosphere. Samples were prepared and weighed into aluminium crucibles (3–10 mg), sealed (with a small, pierced hole to allow for pressure dissipation) and lastly, heated at a rate of 10 °C/minute over a range of 0-300 °C. The thermograms were plotted as a function of heat flow against temperature. Degradation temperatures of the samples were determined using the TGA as the percentage weight change over a temperature range of 30–900 °C with a heating rate of 10 °C/minute. Samples were maintained in an inert N_2_ environment and degradation thermograms were plotted as percentage weight against temperature.

#### Crystallinity analysis

The crystallinity of the samples was determined using a benchtop MiniFlex 600 X-Ray Diffractometer (Rigaku, Tokyo, Japan). The pure polymers, the lyophilised unloaded and loaded nanoparticles, and the lyophilised unloaded and loaded hydrogels were finely packed onto the sample holders with double-sided tape. CuKα radiation (15 mA, 40 kV) was employed on the samples which underwent a 2θ scan range between 5–60° at a rate of 5°/minute.

### Viscoelastic measurements of the hydrogel

The viscoelastic properties of the loaded and unloaded hydrogels were determined using the ThermoHaake MARS Modular Advanced Rheometer (Thermo Fischer Scientific, Karlsruhe, Germany) pre-equipped with a temperature-controlled bath allowing for temperature control of the sample chamber. Strain sweep, shear rate ramp, frequency sweep and temperature ramp measurements were conducted. All measurements were conducted in triplicate using a parallel plate with a gap measurement of 0.052 mm and measuring geometry of C35/1° T_1_.

Hydrogel formation in the simulated vitreous humour (SVH) was evaluated using the ElastoSens™ Bio2 (Rheolution, Montreal, Quebec, Canada). The real-time in situ formation of both hydrogels was evaluated in triplicate to assess the soft mechanical properties of each hydrogel. The experiment was conducted by placing 3 mL of formulated hydrogel (unloaded and loaded) in 1 mL of SVH to mimic the in vitro environment during administration. The shear storage (G’) and loss (G”) modulus of both hydrogels was measured as a function of time at a constant temperature of 37 °C.

The injectability of the hydrogels with a 26-gauge (26G) and 31-gauge (31G) needle was studied using the TA-XT2 Texture Analyser (Stable Micro Systems, Surrey, UK). All measurements were conducted in triplicate with the sample temperature at 10 °C (refrigerator storage temperature) in compression mode. A needle (26G or 31G) was fitted into a 3 mL luer-lock syringe and placed vertically in the holder with the needle facing downwards. Manual syringe injection was mimicked using a P/50R cylinder probe aligned to the plunger plate for volume displacement. Compression of the syringe plunger using the probe (compression speed of 1 mm/s and distance of 20 mm) expels the hydrogel from the syringe and the maximum force was recorded [86, 87].

### Optical properties of the hydrogel

The refractive index of the unloaded and loaded hydrogels was measured in triplicate using the Anton Paar refractometer (Anton Paar OptoTec GmbH, Germany) and the transmittance of the hydrogels was determined using the Implen Nanophotometer (NP80, UV//Vis spectrophotometer, Munich, Germany). The transmittance of the hydrogels was measured at 37 °C at a wavelength range of 200–950 nm.

### In vitro drug release studies

In vitro drug release analysis was undertaken using dialysis membranes in a simulated vitreous humour (SVH) buffer solution (4 mL). Analysis was conducted on drug release from the nanoparticle-loaded hydrogel as well as the drug-loaded nanoparticles alone until an equilibrium release state was reached.

An orbital shaker incubator was used to incubate all samples for the duration of the drug release studies at a constant temperature of 37 °C. Samples of 1 mL were withdrawn at each time point for analysis and replaced with 1 mL of fresh SVH solution to maintain sink conditions. Samples were analysed using a spectrophotometer (Lambda 25 UV/Vis spectrophotometer, PerkinElmer, MA, USA) in the same conditions used for the preparation of the calibration curve. The concentration of triamcinolone acetonide released was determined using a calibration curve constructed from absorbance measurements of known drug concentrations.

### Swelling behaviour, degradation profile determination and stability

The swelling behaviour of the unloaded and loaded hydrogel was studied with all experiments conducted in triplicate. The swelling ratio of the hydrogels was measured by the immersion of the hydrogels in SVH at 37 °C in an orbital shaker. The weights of the swollen hydrogels (W_S_) were measured every hour until equilibrium was reached and each sample was allowed to dry (W_D_). The swelling ratio was calculated according to Eq. 2, and the equilibrium ratio was determined as the ratio at which no further change in hydrogel mass occurred.

2 $$ Swelling\, ratio= \frac{{W}_{S}-{W}_{D}}{{W}_{D}}$$

The degradation profiles of the unloaded and loaded hydrogel were determined with experiments conducted in triplicate. The degradation of the hydrogels was determined by immersing the hydrogel in SVH with lysozyme solution (10,000 U/mL) at 37 °C in an orbital shaker. The weights of the hydrogels were measured at a specific time point for 60 days. The lysozyme-SVH was replaced every 48 h. Degradation of the hydrogels was determined using Eq. 3.

3 $$ Degradation \left(\%\right)= \frac{{W}_{t}- {W}_{0}}{{W}_{0}} \times 100$$

Where W_0_ is the initial weight of the hydrogels prior to immersion and W_t_ is the weight of the hydrogels at each time point after immersion.

To assess the stability of the unloaded and loaded hydrogels, they were sealed in glass vials and stored at cold storage temperature (5 °C ± 3 °C) for 3 months. At the end of one month and the end of the storage period, the hydrogels were assessed for physical appearance, viscosity, refractive index, pH, in vitro gelation time, and in vitro drug release.

### In vitro cell biocompatibility

Biocompatibility of the drug and the unloaded and loaded hydrogels was evaluated in an NIH/3T3 (mouse fibroblast) (ATCC) and an hRPE (human retinal pigment epithelium) (ATCC) cell culture. NIH/3T3 cell maintenance was achieved in Dulbecco’s Modified Eagle Medium (DMEM), with 10% Fetal Bovine Serum (FBS) and 1% penicillin/streptomycin antibiotic at 37 °C, 95% humidity and 5% CO_2_. hRPE cell maintenance was achieved in DMEM/Hams F12 containing 10% FBS and 1% penicillin/streptomycin antibiotic at the same environmental conditions as the NIH/3T3 cell line. Culture mediums were replaced every 2 days until cell confluence (90%) was reached. Cell lines were seeded with a seeding density of 5 × 10^4^ cells/mL.

The cells were treated with the sterile hydrogels (0.5 mL) and the drug solution (exposed to UV sterilisation for 12 h) at a concentration corresponding to the administered TA dose (~ 4 mg) in the culture medium and incubated for 48 and 72 h in 96-well plates at 37 °C, 95% humidity and 5% CO_2_. An MTT cell viability assay was conducted using an MTT-based Roche Cell Proliferation kit. MTT solution (10 µL) was added to each well and the plates were incubated at 37 °C, 95% humidity and 5% CO_2_ for 4 h. Thereafter, 100 µL MTT solubilising agent was added to dissolve the formazan crystals overnight. A multi-plate reader (BioTek, USA) was used to determine the absorbance of the plates at 570 nm. The relative cell viability was calculated using Eq. 4.

4 $$ Cell\, Viability \left(\%\right)= \frac{{Abs}_{S}}{{Abs}_{C}} \times 100$$

Where Abs_S_ is the absorbance of the sample and Abs_C_ is the absorbance of the control (no treatment).

### Pilot in vivo drug release and biocompatibility evaluation in a rabbit model

In vitro results of toxicity, biodegradation and cell behaviour are known to vary under in vivo settings [88]. In vivo analysis enables the assessment of the clinical translational potential of the fabricated nanoparticle-loaded hydrogel under in vivo conditions. To assess the full safety of the hydrogel an in vivo assessment is essential. In vivo pilot studies allow for the initial assessment of the biocompatibility of therapeutic agents while minimising the number of animals used.

Studies on the vitreous of various animals have been conducted to assess their nature and composition in relation to the human vitreous [4, 21, 89]. These studies have shown that the vitreous humour of the rabbit has many similarities to the human vitreous. Additionally, the rabbit model has been used for various ophthalmic surgery evaluations as the rabbit eye is larger than the rodent eye and, thus, the ocular tissues, including the vitreous, are larger allowing for easier surgical procedures [90–92]. This is especially advantageous for surgeries involving the posterior segment of the eye. In vivo studies of various intravitreal injections as well as pars plana vitrectomies with polymeric vitreous substitutes have been conducted using the New Zealand White (NZW) rabbit model [51, 93–97]. Therefore, established protocols and data are available for comparative analysis to determine the efficacy of the fabricated hydrogel. Thus, NZW rabbits were used as the animal model for this study.

This pilot study served as an initial exploratory study for assessing the release potential of the loaded hydrogel and well as its safety. The unloaded hydrogel was not assessed as the purpose of the pilot study was to assess the preliminary drug release behaviour and biocompatibility of the entire system (nanoparticle-loaded hydrogel), while limiting animal numbers. There is no clinically approved vitreous substitute that provides TA release for comparison. Following success of the approved pilot study, a subsequent full preclinical investigation would be undertaken and reported, with inclusion of a placebo, and monitoring of inflammatory changes and transitions in intraocular pressure (IOP). This study thus assessed the biocompatibility and in vivo drug release profile of the hydrogel over 28 days.

### Animal procurement

The New Zealand White (NZW) rabbits (male and female, weight 2.5–4.5 kg) utilised in this study were procured from the Wits Research Animal Facility (WRAF) of the University of the Witwatersrand. Ethics clearance for this study was obtained from the Wits Animal Ethics Screening Committee, ethics clearance number: 2021/03/07/C.

### Animal preparation and care

Fifteen NZW rabbits of both sexes were used for the pilot in vivo study involving intravitreal vitreous removal, vitreous replacement, and subsequent biocompatibility testing. NZW rabbits were individually housed, and cages were maintained at room temperature (25 °C). The rabbits were fed a commercial diet and water was given ad libitum. The NZW rabbits were allowed to acclimatise to the environment one week before the surgery and weighed weekly. A weight loss of 15% or more of its body weight would result in the removal of the rabbit from the study. All procedures conformed to the South African standard for the use and care of animals for scientific purposes and were conducted following the standard operating procedures (SOPs) of the WRAF.

### Experimental design and surgical protocol

Rabbits were grouped according to their similarity in weight in groups of three for each time point (Days 5, 7, 14, 21 and 28). Surgery and intervention were conducted in the left eye and the right eye of each rabbit would serve as the negative control (no intervention). Each rabbit was injected with 500 µL of the formulated hydrogel after the removal of the same amount of native vitreous with the procedure discussed henceforth.

An ophthalmological examination of the rabbit eyes was conducted prior to the surgery to ensure that no ocular abnormalities were observed. General anaesthesia was induced with an intramuscular (IM) combination of ketamine and xylazine (40 mg/kg and 10 mg/kg respectively). A wire speculum was inserted in the left eye after which the eye was washed with several drops of 5% povidone/iodine and 0.2 mL lignocaine, local anaesthetic, was instilled. A 26-gauge needle and 1 mL syringe were used to puncture the eye, 2.0 mm posterior to the superior-temporal limbus where the needle would be visible in the pupil area. Vitreous humour (500 µL) was gently aspirated after which the syringe was replaced with a 1 mL syringe containing 500 µL of the loaded hydrogel. The needle and syringe were held in place for about 30 s to allow for the hydrogel to increase in viscosity, to prevent reflux, and to ensure no vitreous haemorrhage occurred. Once the needle was withdrawn, direct pressure was placed on the site of injection. All formulations were sterilised via UV sterilisation overnight. An anaesthetic reversal agent was administered IM to each rabbit, and they were then placed in their cage for recovery. Following surgery, qualitative cage-side observations were conducted following the rabbit grimace scale [98] and their weight was measured weekly.

### Euthanisation, enucleation and sampling

Three NZW rabbits were euthanised at each time point (day 5, day 7, day 14, day 21 and day 28). Before euthanisation, 1 mL of blood was taken from each rabbit and centrifuged (Eppendorf centrifuge 5804, Hamburg, Germany) for blood serum drug release studies. An overdose of IV sodium pentobarbital (> 50 mg/kg, 2 mL) was administered via the ear marginal vein for euthanisation followed by enucleation (removal of the eyeball) of the control eye (right eye) and the treated left eye. Aqueous humour and vitreous humour were aspirated from the treated eye and immediately frozen and maintained at -80 °C for analysis. For vitreous humour sampling, the needle was positioned close to the posterior pole of the globe to limit sampling of the actual vitreous substitute (which was injected posterior to the superior-temporal limbus, and thus would be positioned in the anterior portion of the vitreous chamber due to rapid thermoresponsive gelling on injection), and maximise sampling of the native vitreous humour (which also forms a larger volume proportion of the vitreous chamber). The remaining enucleated ocular tissue was immersed and fixed in 10% ^v^/_v_ buffered formalin for histomorphological analysis.

### Histomorphological analysis

The enucleated eyes (fixed in 10% ^v^/_v_ formalin) were sent for analysis to IDEXX laboratories (Johannesburg, South Africa). The eyes were cut according to the IDEXX SOP (IdexxSA-AP-SOP-26) and processed in an automated tissue processor according to the histological tissue processing SOP (IdexxSA-AP-SOP-27). Thereafter, sections of the tissue were cut (IdexxSA-AP-SOP-30) and the slides produced stained in an automated Haematoxylin and Eosin tissue stainer (IdexxSA-AP-SOP-205) prior to histological evaluation.

Assessment of the level of inflammation was histopathologically graded to ascertain the propensity of hydrogel to induce an ocular inflammatory response; the infiltration of inflammatory cells into the vitreous cavity was visualized where Grade 0 = absent, Grade 1 = mild, Grade 2 = moderate, and Grade 3 = severe. Cellular infiltration (degree of infiltration by lymphocytes, plasma cells, and mononuclear and polymorphonuclear leukocytes) was graded from 0 to 3 + in accordance with IDEXX laboratory protocols.

### In vivo drug release studies

Aspirated and frozen blood serum, aqueous humour, and vitreous humour were thawed and centrifuged (Eppendorf centrifuge 5804, Hamburg, Germany). To ensure that the drug levels detected within the removed aqueous and vitreous samples were only that of unencapsulated drug (i.e. free drug released from the nanoparticles), samples were centrifuged allowing for drug-loaded nanoparticles to be separated from the supernatant, which was subsequently analysed. The concentration of TA was evaluated by UV-Vis (Implen Nanophotometer NP80, Implen, München, Germany) at a wavelength of 240 nm as established previously. The absorbance readings were analysed via the calibration curve to determine the release of TA.

### Statistical analysis

All analyses were conducted in triplicate (*n* = 3). All results were expressed as the mean and standard deviation and data was analysed using Microsoft EXCEL. The level of significance was determined using a student t-test. P values of less than 0.05 (*p* < 0.05) were considered as an indication of statistically significant differences. All graphs were plotted using SigmaPlot 12.0.

## Results and discussion

**Fig. 1:**
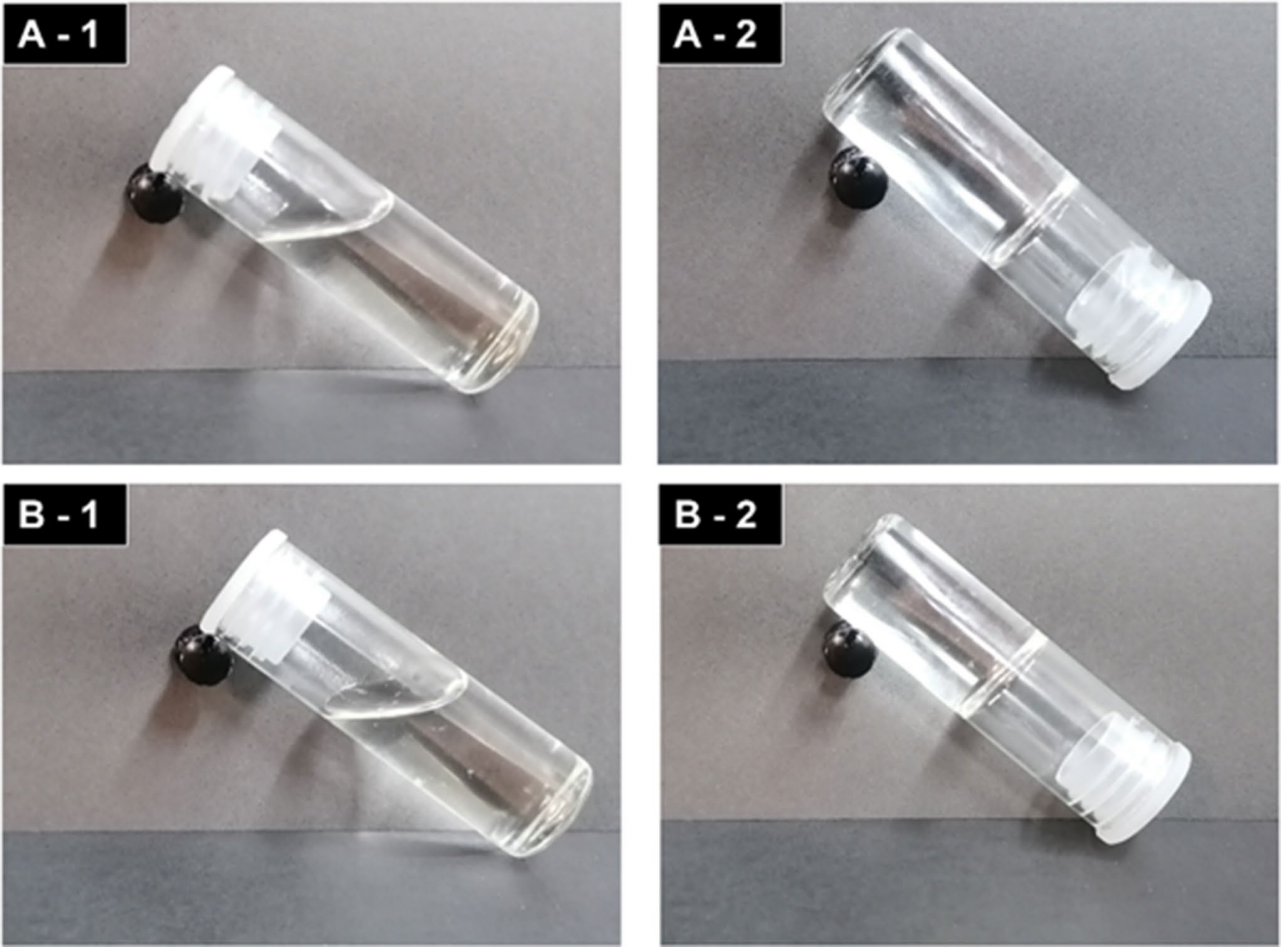
Sol-gel phase transition with the vial inversion method at physiological temperatures. (**A**) Sol-gel transition of the unloaded hydrogel (**B**) Sol-gel of the nanoparticle loaded hydrogel

A nanoparticle-loaded hydrogel was successfully fabricated. An optically transparent and clear soft-gel (Fig. 1A and B) was obtained that underwent sol-gel phase transition at physiological temperatures. Rheological data, discussed extensively in Section “Viscoelastic measurements of the hydrogel”, is also indicative of the sol-gel transition at physiological temperatures for the unloaded (33.688 °C ± 0.021 °C) and loaded hydrogels (36.496 °C ± 0.014 °C).

Gel formation was observed to occur at ~ 33 °C for the unloaded hydrogel and ~ 36 °C for the nanoparticle-loaded hydrogel. The temperature of gel formation for the nanoparticle-loaded hydrogel is favourable as it is similar to physiological temperatures. Gel formation of the nanoparticle-loaded hydrogel occurred at a higher temperature than that for the unloaded hydrogel due to interactions between the nanoparticles and the micelles formed during the sol-gel transition. A higher temperature is needed to overcome these interactions for gel formation. The time taken for gel formation at 37 °C from 10 °C of the unloaded hydrogel and the loaded hydrogel were measured. These times of nine minutes (± 0.6 min) for the unloaded hydrogel, and eight minutes (± 0.2 min) for the loaded hydrogel, are clinically advantageous. Vitreous substitution with current tamponades requires patients to remain face-down post-vitrectomy [2, 16]. This fabricated hydrogel would form almost immediately post-vitrectomy increasing patient compliance.

### Nanoparticle size, surface charge, surface morphology, drug encapsulation and hydrogel loading

Nanoparticles formed were screened to have a diameter of < 200 nm with a PDI of ≤ 0.2. Previously, PLGA nanoparticles for ocular administration (< 200 nm) were prepared for lipophilic drugs using the solvent-evaporation method [99, 100]. Various solvents have been used for the fabrication of PLGA nanoparticles and PLGA nanoparticles for ocular administration utilising acetone, ethyl acetate and dichloromethane [101]. Acetone was not used in this study as particles formed with acetone would likely yield larger particles than those formed with dichloromethane due to the water miscibility of acetone [102]. Dichloromethane is reported to be a favourable solvent in the formation of PLGA nanoparticles [103, 104], however, due to the cytotoxic nature of dichloromethane, ethyl acetate was used to decrease the concentration of dichloromethane in the formulation. Table 1 includes the various solvent ratios used and shows that lower dichloromethane concentrations with ethyl acetate are favourable for the fabrication of nanoparticles with a size < 200 nm and a PDI < 0.2. The negative surface charge observed for all the nanoparticle formulations is attributed to the carboxylic acid end groups of PLGA.

**Table 1:** Size, dispersity, and surface charge of PLGA nanoparticles in various solvent ratios

PVA concentration	Organic mixture ratio (EA: DCM)	Particle size (diameter) (nm)	PDI	Particle surface charge (mV)
1% ^w^/_v_	1:1	184 ± 4.1	0.205 ± 0.035	-31.3 ± 0.8
	1:2	245 ± 2.6	0.247 ± 0.005	-43.5 ± 0.2
	1:3	235 ± 3.4	0.263 ± 0.003	-26.4 ± 0.4
	2:1	145 ± 2.5	0.187 ± 0.020	-26.3 ± 0.1
	3:1	134 ± 1.0	0.155 ± 0.006	-25.1 ± 0.5

Nanoparticles with a solvent ratio of 2:1 and 3:1 (ethyl acetate: dichloromethane) both had the necessary particle size and PDI. Thus, to identify the preferred formulation for entrapment within the hydrogel, the encapsulation efficiency with triamcinolone acetonide (TA) of both formulations was tested. The formulation with a 3:1 ratio of ethyl acetate: dichloromethane had a higher drug encapsulation of 97.500% (± 1.523%) while encapsulation within the 2:1 formulation was 78.123% (± 2.271%). The PLGA nanoparticle formulation with a 3:1 solvent ratio (ethyl acetate: dichloromethane) was chosen for encapsulation within the hydrogel.

**Fig. 2:**
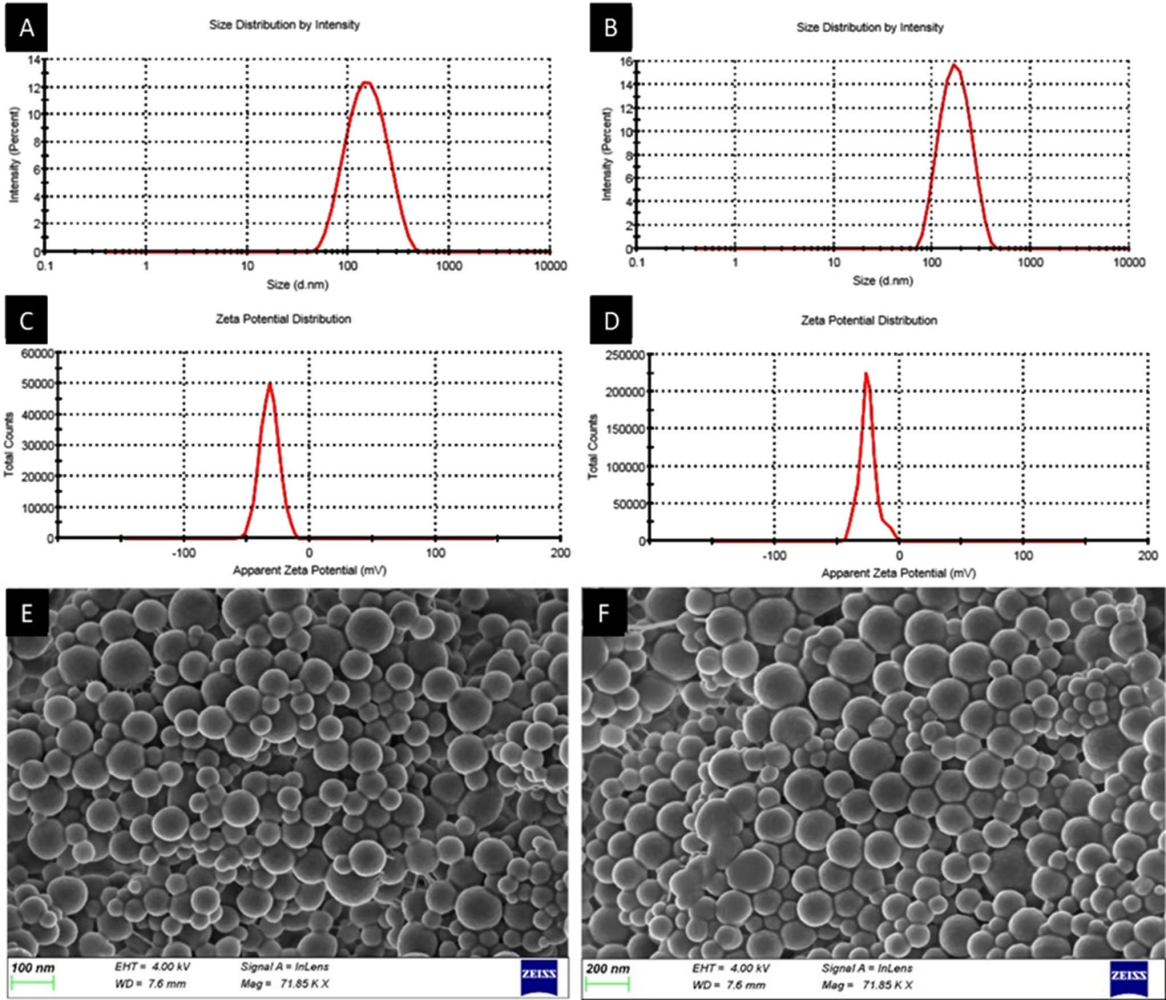
Zeta analysis of the unloaded and loaded nanoparticles. **A**, **B** Particle size distribution (mean ± S.D., *n* = 3) and **C**, **D** Zeta potential distribution of the: **A**, **C** unloaded and **B**, **D** TA loaded PLGA nanoparticles (all experiments were conducted in triplicate, *n* = 3, and reported as the mean ± S.D. Representative data is shown). **E**,**F** SEM micrographs of the **E** unloaded PLGA nanoparticles and **F** TA-loaded PLGA nanoparticles

The particle size and PDI of the unloaded nanoparticles (Fig. 2A) and the TA-loaded nanoparticles (Fig. 2B) were 133.7 ± 1.0 nm (PDI = 0.155 ± 0.006) and 153.1 ± 2.4 nm (PDI = 0.179 ± 0.020), respectively. The surface charge of both the unloaded and loaded nanoparticles was negative due to the negative charge of PLGA (Fig. 2C & D).

SEM micrographs (Fig. 2E & F) show the spherical morphology of the fabricated nanoparticles. Slight particle size increases are noted between the unloaded nanoparticles (Fig. 2E) and the drug-loaded nanoparticles (Fig. 2F). The particles are visualised to be dispersed with no drug crystals visible unlike those noted by Gómez-Gaete and co-workers [105]. The lack of drug crystals shows entrapment of the drug and this is anticipated to eliminate the burst release of the drug from the nanoparticles as reported in previous PLGA nanoparticle studies [100, 105, 106].

As discussed, drug loaded nanoparticles were lyophilised and resuspended to formulate the loaded hydrogel (4 mL). For both in vitro and in vivo analysis drug release analyses, the final nano-enabled hydrogel contained 48.7 mg ± 0.168 mg of nanoparticles and a dose of 3.9 mg ± 0.036 mg of TA in 500 µL of the hydrogel.

### Physicochemical properties of the nanoparticles and the hydrogel

**Fig. 3:**
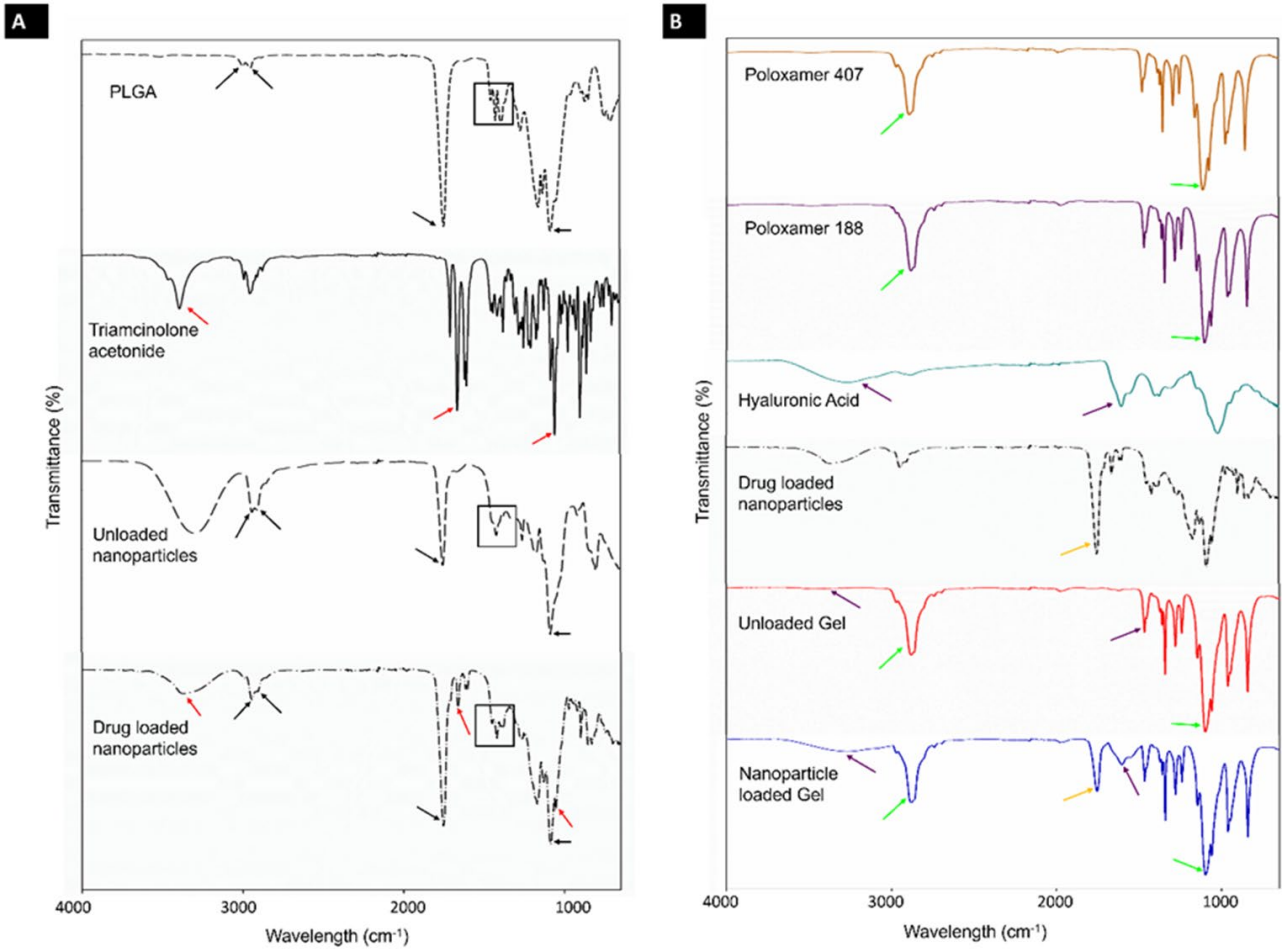
FTIR spectra of (**A**) PLGA, triamcinolone acetonide and the unloaded and loaded nanoparticles; (**B**) Both poloxamers, hyaluronic acid, the loaded nanoparticles, and the unloaded and loaded hydrogels

FTIR spectra of the PLGA nanoparticles, native triamcinolone acetonide, and PLGA are shown in Fig. 3A. Triamcinolone acetonide (TA)-loaded nanoparticle fabrication was confirmed by FTIR. Unloaded and TA-loaded PLGA nanoparticles displayed IR absorption bands characteristic of the PLGA polymer (indicated by the black arrows). These bands include C-H bending at 3002 cm^− 1^ and 2956 cm^− 1^, the C-O ester double bond at 1760 cm^− 1^, and C-O stretching at 1091 cm^− 1^. Lactide-lactide (1456 cm^− 1^), glycolide-glycolide (1429 cm^− 1^) and lactide-glycolide (1394 cm^− 1^) bands of the PLGA monomers were also visible as a triple-peak (designated by the black box).

The FTIR spectrum of pure TA showed the characteristic H-bond hydroxyl stretching IR absorption band at 3398 cm^− 1^, carbonyl stretching at 1706 cm^− 1^ and C-F stretching at 1057 cm^− 1^. These TA IR absorption bands (indicated by red arrows) are also visible in the TA-loaded nanoparticles. Polymer and drug interactions did not take place as new peaks were not visible and shifts in existing peaks were not observed. The presence of these peaks suggests that TA was successfully encapsulated in the PLGA nanoparticles.

FTIR spectra of the hydrogels and their polymers are shown in Fig. 3B. Poloxamer 407 and Poloxamer 188 displayed similar FTIR profiles due to their similarities in their composition. Both showed characteristic hydroxyl stretching bands at 3400 cm^− 1^ and C-O-C stretching at 1085 cm^− 1^. These bands (indicated by the light green arrows) were also prominent in the final unloaded and nanoparticle-loaded hydrogels. The FTIR spectrum of HA displays characteristic IR absorption bands at 3318 cm^− 1^ (hydroxyl stretching), C-O-C stretching at 1151 cm^− 1^ and 1024 cm^− 1^, amide bands at 1530 cm^− 1^ and 1565 cm^− 1^, and carboxylic bands at 1605 cm^− 1^ and 1405 cm^− 1^. HA hydroxy groups and a carboxylic band are visible in the unloaded and loaded nanoparticle spectra (purple arrows) indicative of the presence of HA in the hydrogels. The nanoparticle-loaded hydrogel also displays a peak at 1760 cm^− 1^ (yellow arrow) indicative of a C-O ester bond attributed to the PLGA within the nanoparticle system. HA and nanoparticle bands in the hydrogels had lower transmittance values due to the high amount of poloxamer in comparison to HA. Similar results have also been observed in physically mixed poloxamer gels [107]. Shifts in peaks were not visible and new peaks were absent. This confirms that chemical interactions between the hydrogel and nanoparticles did not take place.

DSC analysis was performed on the unloaded and loaded PLGA nanoparticles, TA, and PLGA (Supplementary Fig. 1). PLGA displayed an endothermic peak at 51.14 °C indicative of its glass transition [108]. PVA displayed an endothermic peak at 227.84 °C associated with the melting point. This endothermic peak was further visible in the unloaded and loaded PLGA nanoparticles. A sharp endothermic peak at 295.87 °C was observed for TA due to the melting point of the crystalline state of TA [109]. This sharp endothermic peak was not visible in the TA-loaded PLGA nanoparticles, which may indicate a shift to a more amorphous form leading to increased solubility of the drug in the nanoparticle [109, 110] or due to solid state interaction by heating [111, 112].

The DSC thermograms of the hydrogels and their constituents are shown in Supplementary Fig. 1. The DSC thermograms of Poloxamer 407 and Poloxamer 188 displayed endothermic peaks at 57.66 °C and 55.27 °C, respectively. These are indicative of the melting temperature of poloxamers. The unloaded and loaded hydrogels displayed the same peak. However, these peaks had slight shifts in comparison to the pure polymers. This would be a result of the physical mixing of HA and the nanoparticles in the hydrogels. The DSC thermogram of HA displays a gentle endothermic peak at 133 °C and a steep exothermic peak at 238 °C indicative of the degradation of HA. This exothermic peak is visible in the unloaded and loaded hydrogel thermograms. The PLGA nanoparticle degradation is also visible in the thermogram of the loaded hydrogel. The high concentration of poloxamer, as noted in the FTIR spectra, masks peaks that were visible in the thermogram of the nanoparticles.

TGA thermograms (Supplementary Fig. 2) were subsequently correlated to the DSC thermograms. Complete degradation of all prepared formulations (nanoparticles and hydrogels) occurred with major weight loss occurring between 270 and 500 °C for both nanoparticle formulations, and between 375 and 500 °C for both hydrogel formulations. The degradation of the nanoparticle formulations is congruent with the DSC thermograms where the onset of an endothermic peak is visible towards 300 °C in the thermograms. The TGA thermograms also display a decreased degradation rate for the nanoparticle-loaded hydrogel in comparison to the unloaded hydrogel.

The XRD patterns of the prepared nanoparticle formulations, TA, and the pristine polymers are shown in Supplementary Fig. 3. PLGA and PVA displayed amorphous surface characteristics while TA showed various crystalline peaks. The unloaded nanoparticles display an amorphous structure similar to the structure observed with pure PLGA. An amorphous structure is also visible for the drug-loaded nanoparticles; however, some TA peaks were visible. This amorphous structure of the loaded nanoparticles is attributed to the decreased drug crystallinity and solubilisation in PLGA during the synthesis of the nanoparticles.

The XRD pattern of the unloaded hydrogel is very similar to that of the pure poloxamer polymers. However, differences in structure are visible between 23° and 29°. The loaded hydrogel displays characteristics similar to the unloaded hydrogel except for changes visible between 30° and 54°. The changes in the unloaded hydrogel are attributed to HA while deviations in the loaded hydrogel are due to the presence of the nanoparticles. The intensity of these variations is low, purportedly due to the lower HA and nanoparticle concentrations in comparison to that of the poloxamers.

### Viscoelastic measurements of the hydrogel

**Fig. 4:**
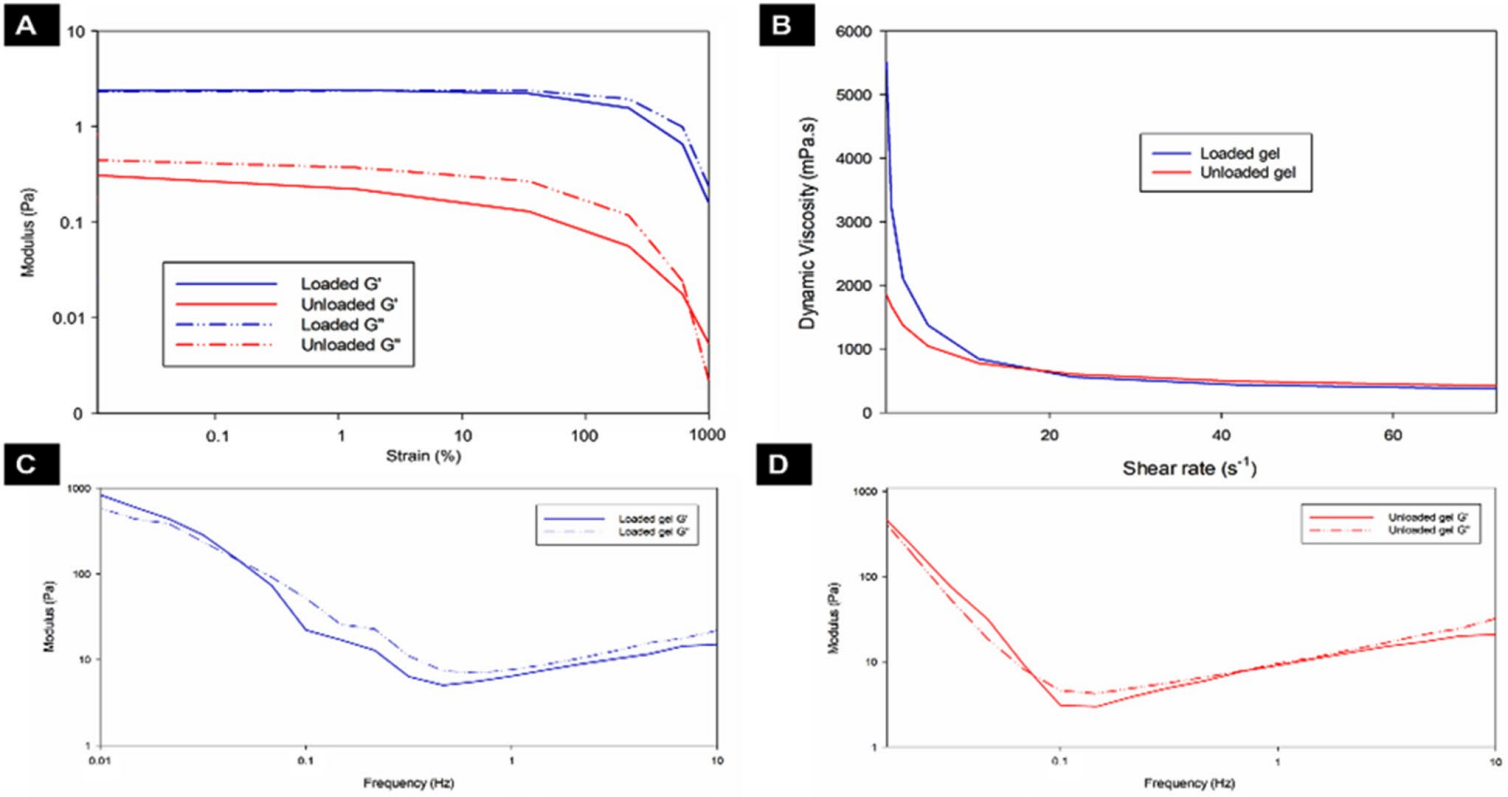
Rheological data for the unloaded and loaded hydrogels. (**A**) Strain sweep experiments to determine LVR, (**B**) Shear rate ramp experiments, (**C**) Frequency sweep of the loaded hydrogel and (**D**) Frequency sweep of the unloaded hydrogel at 37 °C (all experiments were conducted in triplicate, *n* = 3, and reported as the mean ± S.D. Representative figures are shown)

The unloaded and loaded hydrogels displayed similar viscoelastic properties to the natural vitreous. It is necessary to determine the linear viscoelastic range (LVR) to further understand the linear behaviour of the hydrogels. A strain sweep analysis was conducted to ascertain the LVR of both hydrogels. Figure 4A shows that the LVR for both hydrogels was below a strain of 10%. The study was conducted at a constant frequency of 1 Hz as variations in modulus are easily detected at this frequency. G’ (storage modulus) and G” (loss modulus) decreased above a 10% strain and the loss modulus for the loaded hydrogel became larger than the storage modulus. This suggests that the hydrogel became more liquid. As a result, a 1% strain was used to conduct all subsequent rheology studies on both hydrogels.

Shear thinning of both hydrogels was observed with a decrease in viscosity as the shear rate increased (Fig. 4B). This behaviour is ideal for extrusion via a needle for the administration of the gels.

The storage modulus of the natural vitreous is reported to range from 1 to 7 Pa while the loss modulus is reported to range between 0.3 and 1 Pa [89, 113]. However, these studies were conducted on the in vitro vitreous and do not represent the modulus of the in vivo vitreous. The properties of the human vitreous change after removal from the eye [10] and a study conducted by Zimberlin and co-workers [114] demonstrated that the modulus of the in vivo vitreous is higher than that of the in vitro vitreous. The study by Santhanam et al. [10] shows that a suitable vitreous substitute should have a modulus above 100 Pa. Frequency sweep studies were conducted on both hydrogels at a constant temperature of 37 °C (Fig. 3C & D). Table 2 displays that the average modulus of both hydrogels is > 100 Pa. These modulus values are also favourable for vitreous substitutes as they indicate that the hydrogels would not pass through retinal tears [1].

**Table 2:** Average storage and loss moduli of the unloaded and loaded hydrogels

	Storage modulus (G’) (Pa)	Loss modulus (G”) (Pa)
Loaded hydrogel	132.593 ± 0.320	110.889 ± 0.038
Unloaded hydrogel	99.503 ± 0.024	108.581 ± 0.011

**Fig. 5:**
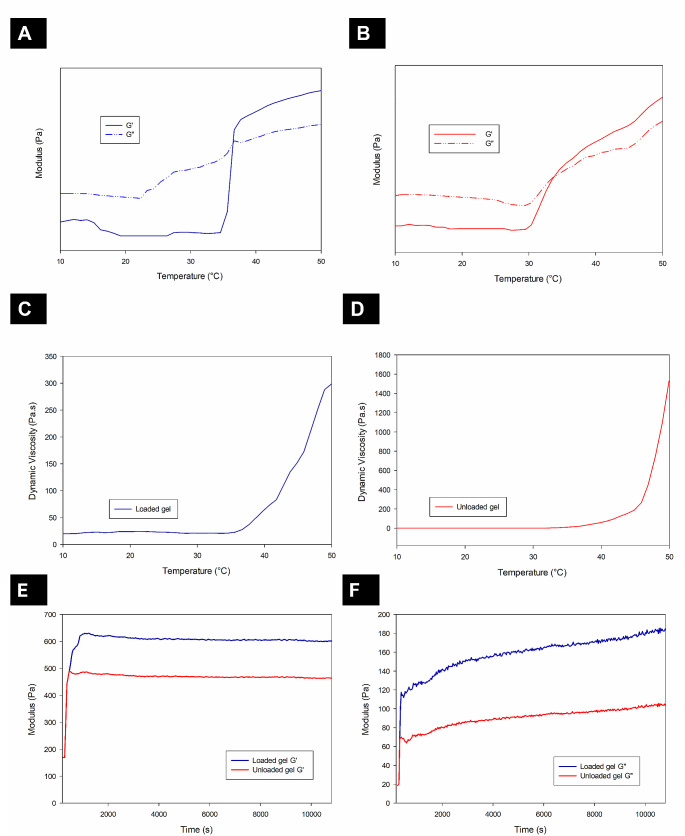
Temperature ramp data of both hydrogels between 10°C and 50°C. (**A**) Modulus of the loaded hydrogel, (**B**) modulus of the unloaded hydrogel, (**C**) viscosity of the loaded hydrogel and (**D**) viscosity of the unloaded hydrogel. **E**, **F** Real-time modulus changes of the unloaded and loaded hydrogels in simulated vitreous humour. (**E**) Storage modulus (G’), (**F**) Loss modulus (G”). (All experiments were conducted in triplicate, *n* = 3, and reported as the mean ± S.D. Representative figures are shown.)

Temperature ramp studies were conducted on both hydrogels to assess their behaviour with changes in temperature (Fig. 5A– D). The storage modulus (G’), loss modulus (G”), and viscosity of the hydrogels were assessed at a temperature range of 10–50 °C. The loaded hydrogel (Fig. 5A & B) displays a cross-over of G’ and G” at 36.496 °C (± 0.014 °C). This cross-over is indicative of the temperature at which the behaviour of the hydrogel becomes that of a gel rather than a solution (gel point). Between temperatures of 33 and 35 °C an increase in G’ and viscosity highlights the commencement of the sol-gel phase change (gel point). This phase change is visible in Fig. 5 (C & D) for the unloaded hydrogel between 29 and 32 °C, with a G’-G” cross-over occurring at a temperature of 33.688 °C (± 0.021 °C). The crossover temperature of the nanoparticle-loaded hydrogel is more favourable compared to the unloaded hydrogel as it occurs close to physiological temperature demonstrating gel formation at physiological temperature.

The ElastoSens™ Bio2 technique for rheological studies measures the real-time properties of the hydrogel in a non-destructive manner. The analysis of the hydrogels is conducted via gentle mechanical vibrations passing through the hydrogel samples that are held within confined holders and the response to the vibrations is detected by a laser. It is important to assess the interaction between the hydrogel (unloaded and loaded) and the native vitreous. Therefore, SVH was prepared to assess this interaction. Figure 5 (E and F) depict the storage modulus (G’) and loss modulus (G”) of both hydrogels in SVH over 3 h. The initial increase of the shear storage modulus and loss modulus of both hydrogels shows the mixing of both hydrogels with the SVH which is evidence of in situ hydrogel formation. The unloaded hydrogel (G’_average_ = 467.01 Pa ± 1.286 Pa) and the loaded hydrogel (G’_average_ = 600.62 Pa ± 2.356 Pa) display properties of soft hydrogels as their modulus was less than 1 kPa. Both hydrogels formed immediately after exposure to the SVH at 37 °C. Maximum gelling and modulus values were achieved in 10 min (± 0.885 min) for the unloaded hydrogel and 18 min (± 1.05 min) for the loaded hydrogel. This was then maintained for the duration of the experiment. These results are for real-time experiments and are thus not directly comparable to those obtained via the rheometer. However, the gelling times are comparable to those observed in the vial inversion test.

The injectability of both hydrogels was conducted with 26-gauge (26G) and 31-gauge (31G) needles. Pars plana vitrectomy is commonly conducted using 23-, 25- and 27-gauge needles [115–117] and intravitreal injections are commonly administered with 26-, 27-, 30- and 31-gauge needles [118–120]. 26G and 31G needles were used for this study. Injection of the hydrogels was possible with both needles; however, a greater force was required for administration with the 31G needle (120.74 *N* ± 0.855 N for the unloaded hydrogel and 97.07 *N* ± 0.584 N for the loaded hydrogel) than with the 26G needle (19.81 *N* ± 0.507 N for the unloaded hydrogel and 17.24 *N* ± 0.696 N for the loaded hydrogel). As pars plana vitrectomy and intravitreal injections utilise 26G needles, and for ease of administration, injection of the hydrogels via 26G needles is preferable.

### Optical properties of the hydrogel

**Fig. 6:**
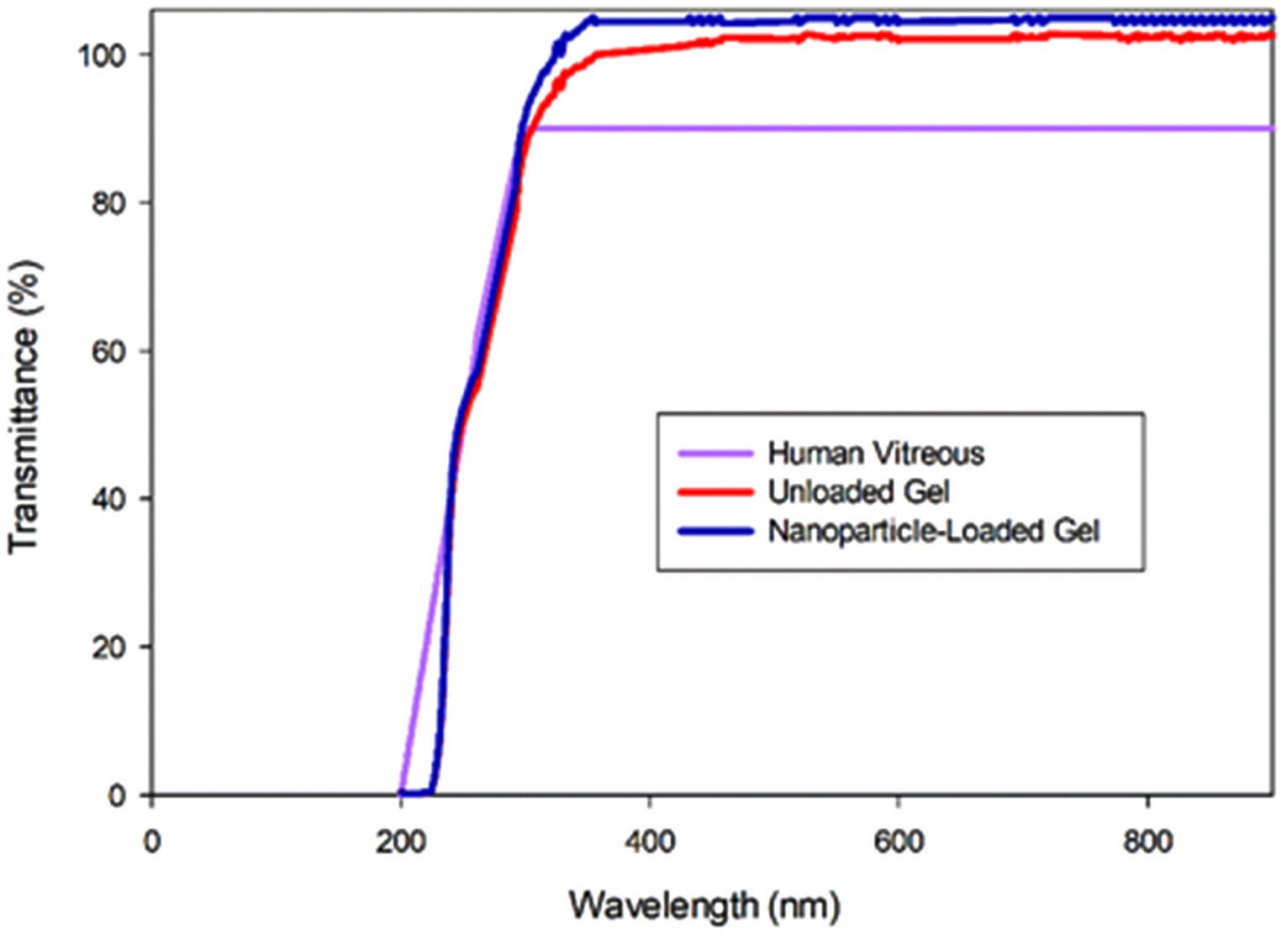
The transmittance of the hydrogels between 200 and 900 nm

The hydrogels had a transparency of > 90% (*p* < 0.05) at 37 °C within the visible wavelengths (Fig. 6). Although the unloaded hydrogel had a higher transmittance than the loaded hydrogel (most probably due to slight nanoparticle interference), both were optically similar to the natural vitreous which allows for 90% of light between 300 and 1400 nm [121]. The transmittance of both hydrogels also behaved similarly to the natural vitreous with a drop in transmittance between a UV range of 0-230 nm.

The refractive index of the unloaded hydrogel was 1.3350 (± 0.002) and the refractive index of the loaded hydrogel was 1.3352 (± 0.003), which are similar to the refractive index of the natural vitreous (1.3349). Silicone oil, the most used vitreous substitute [122], has a refractive index of 1.405 [123]. This has resulted in refractive errors in patients receiving silicone oil as a substitute post-vitrectomy [17].

### In vitro drug release

Drug release studies were conducted to determine the release profile of TA from the PLGA nanoparticles and the nanoparticle-loaded hydrogel. A calibration curve was constructed to determine the release concentrations of TA.

**Fig. 7:**
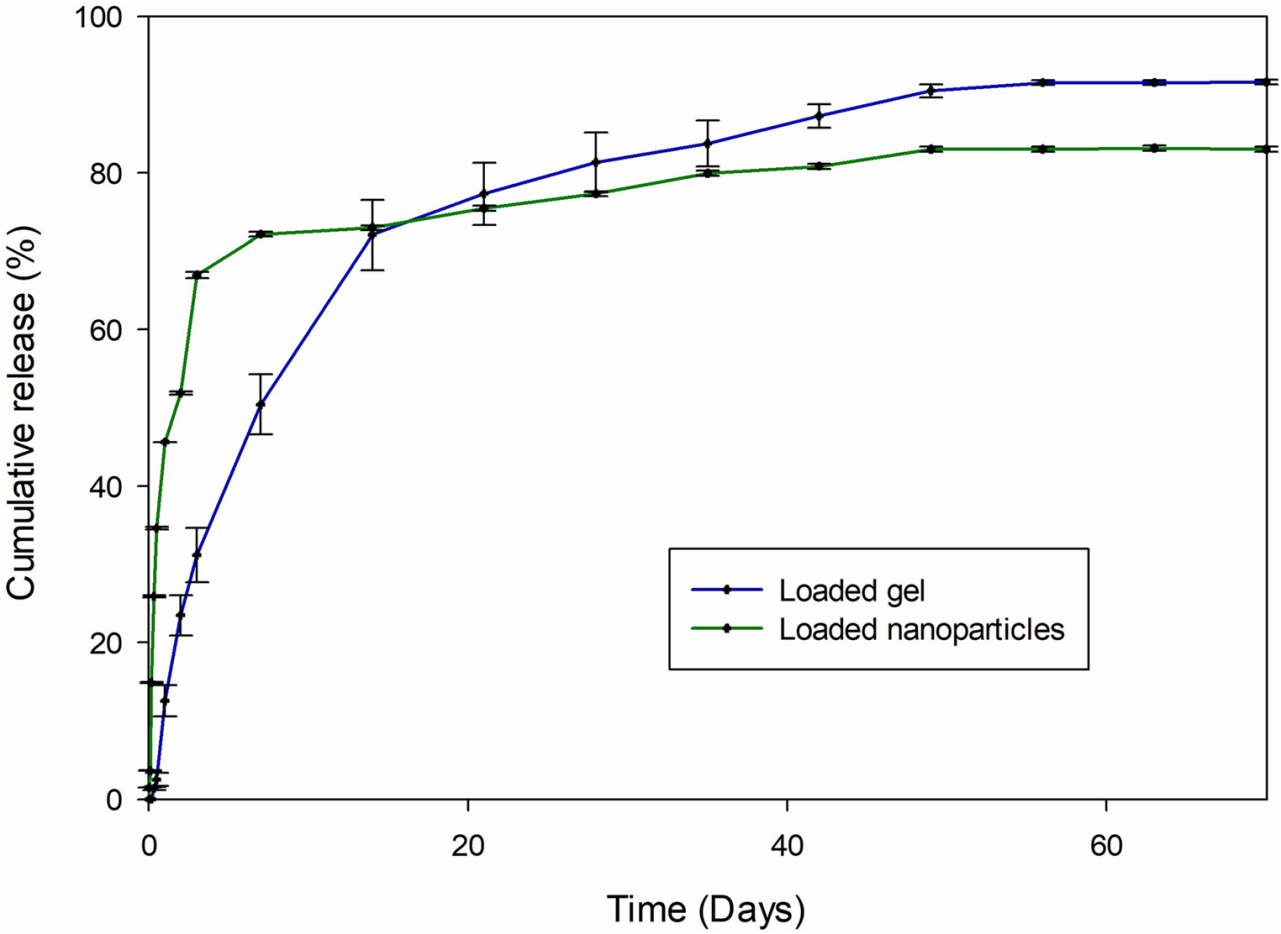
TA release profiles from the nanoparticles and the loaded hydrogel in simulated vitreous humour at 37 °C determined using a spectrophotometer (mean ± S.D., *n* = 3, *p* < 0.05)

Figure 7 shows the in vitro release profiles of TA from the nanoparticles and the nanoparticle-loaded hydrogel, which differed significantly (*p* < 0.05). The drug release profiles show that release of TA from the nanoparticle-loaded hydrogel is slower and over an extended period; whereas the release of TA was initially faster from the nanoparticles alone, which would result in faster clearance of the drug from the vitreous [39].

Half of the encapsulated TA (50%) was released from the nanoparticles within the first day of administration. Comparatively, 50% of TA release from the nanoparticle-loaded hydrogel occurred over the first 7 days. Thereafter there was a slow release of the remaining TA over the remainder of the study period for the nanoparticle-loaded hydrogel, as well as from the nanoparticles alone.

Vitreous substitution can cause inflammation post-vitrectomy [34, 35] and an initial burst release ensued by a slow release of TA from a vitreous substitute would be advantageous for the treatment of the initial acute inflammation. Inflammation due to long-term substitution may also occur and the steady, long-term TA release observed in the loaded hydrogel would allow for treatment of the inflammation. Vitreoretinal diseases such as uveitis, proliferative diabetic vitreoretinopathy, and macular degeneration require treatment with TA. Therefore, TA release from the hydrogel, as well as the biphasic mode of release, is beneficial for inflammation management post-vitrectomy.

### Swelling, degradation and stability

**Fig. 8:**
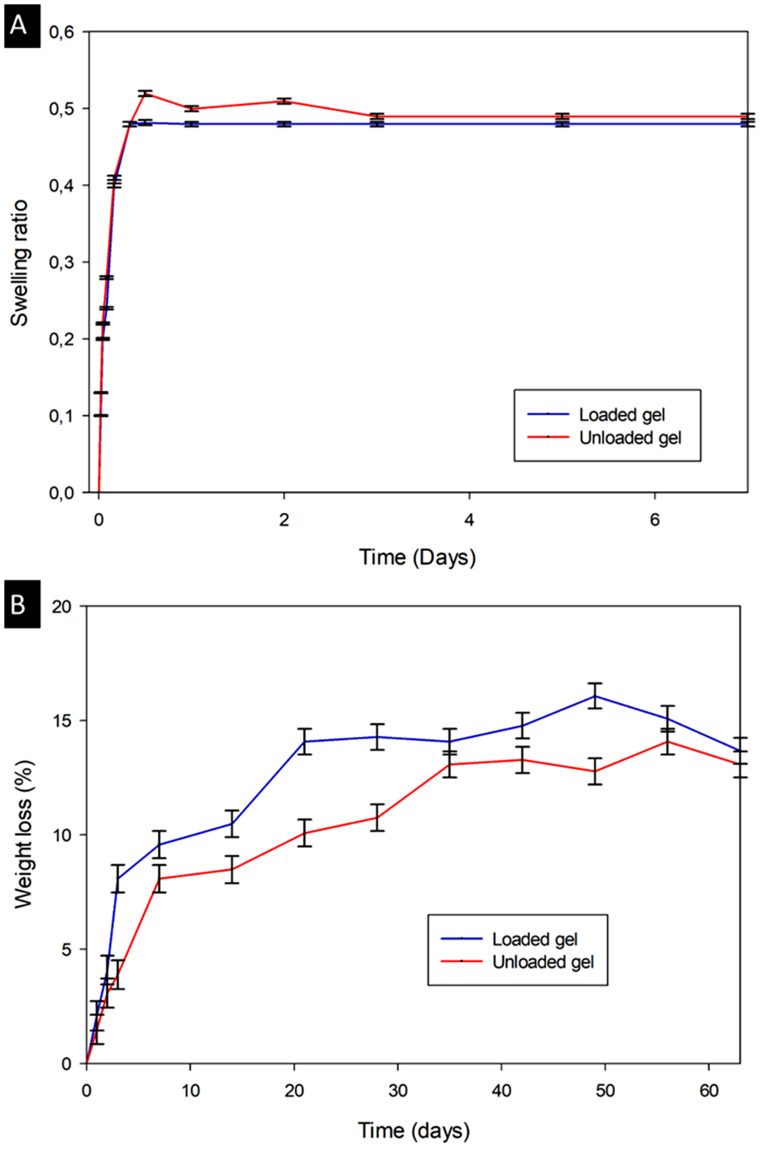
(**A**) Swelling ratio profile of the unloaded and loaded hydrogels in simulated vitreous humour at 37 °C (mean ± S.D., *n* = 3, *p* > 0.05). (**B**) Degradation profiles of unloaded and loaded hydrogels at 37 °C (mean ± S.D., *n* = 3, *p* < 0.05)

The swelling ratio of the unloaded and loaded hydrogels was determined after incubation of the hydrogels in simulated vitreous humour (SVH) buffer solution at 37 °C. This was continued until equilibrium swelling was reached as shown in Fig. 8A. Equilibrium swelling was reached within 12 h for the unloaded hydrogel and eight hours for the loaded hydrogel. The loaded hydrogel reached equilibrium swelling in less time than the unloaded hydrogel due to the presence of the nanoparticles. The total hydrogel volume in the nanoparticle-loaded hydrogel is marginally less, attributed to the decreased time required for equilibrium swelling and the swelling did not exceed 50%. This is advantageous as following the administration of the hydrogel into the vitreous body it should swell rapidly to create the tamponade effect and subsequently result in minimal intraocular pressure (IOP) transitions once equilibrium swelling is attained.

Some swelling of the vitreous substitute is necessary to ensure its ability to tamponade retina on all sides through viscosity and swelling pressure [43]. FDA-approved expandable gases, including SF_6_, and C_3_F_8_, have been employed successfully as short-term vitreous substitutes. SF_6_ increases to twice its volume in 1 to 2 days and lasts for 2–3 weeks. Following injection of pure C_3_F_8_ during surgery, it quadruples its volume in 3 to 4 days and lasts for ~ 6 weeks [1]. Other investigations of polymer-based vitreous substitutes included the preparation of poly(2-hydroxyethyl methacrylate) hydrogel which demonstrated a high swelling of 75%, with good viscoelastic properties [124]. In vivo characterisation is of this system is still required. Vijayasekaran et al. [125] developed two gels for vitreous application including a homopolymer PVP crosslinked with divinyl glycol (DVG) and copolymer of PVP and 2-hydroxyethymlethacrylate (HEMA) crosslinked with the DVG. The preferred hydrogel demonstrated swelling of 98% in addition to the requisite transparency for vitreous applications. The advantage of the nanoparticle-loaded hydrogel developed herein is that equilibrium swelling is rapidly achieved and does not exceed 50% and is anticipated to limit IOP fluctuations.

Biodegradation studies (Fig. 8B) were conducted with the use of lysozyme which acts as a general ocular enzyme at 37 °C. The studies were conducted for up to 63 days to assess the long-term degradation behaviour of both hydrogels. Although a weight loss of the hydrogels was observed, this degradation was less than vitreous substitutes reported by Baker et al. [126] and Yu et al. [127] where the hydrogel mass decreased by 60% in 56 days and 40% in 14 days, respectively. The long-term stability of hydrogels as vitreous substitutes is important to avoid frequent injections and to retard degradation in vitro. Resistance to enzymes as shown with both hydrogels is essential for long-term administration.

Stability of the unloaded and loaded hydrogels was assessed at cold storage temperature (5 °C ± 3 °C) for 3 months. There was no significant difference (*p* > 0.05) in drug concentration, viscosity, refractive index, pH and gelation time before and after cold storage. It is, therefore, recommended that the hydrogels (unloaded and loaded) be stored in cold storage (refrigerated).

### In vitro cell biocompatibility

**Fig. 9:**
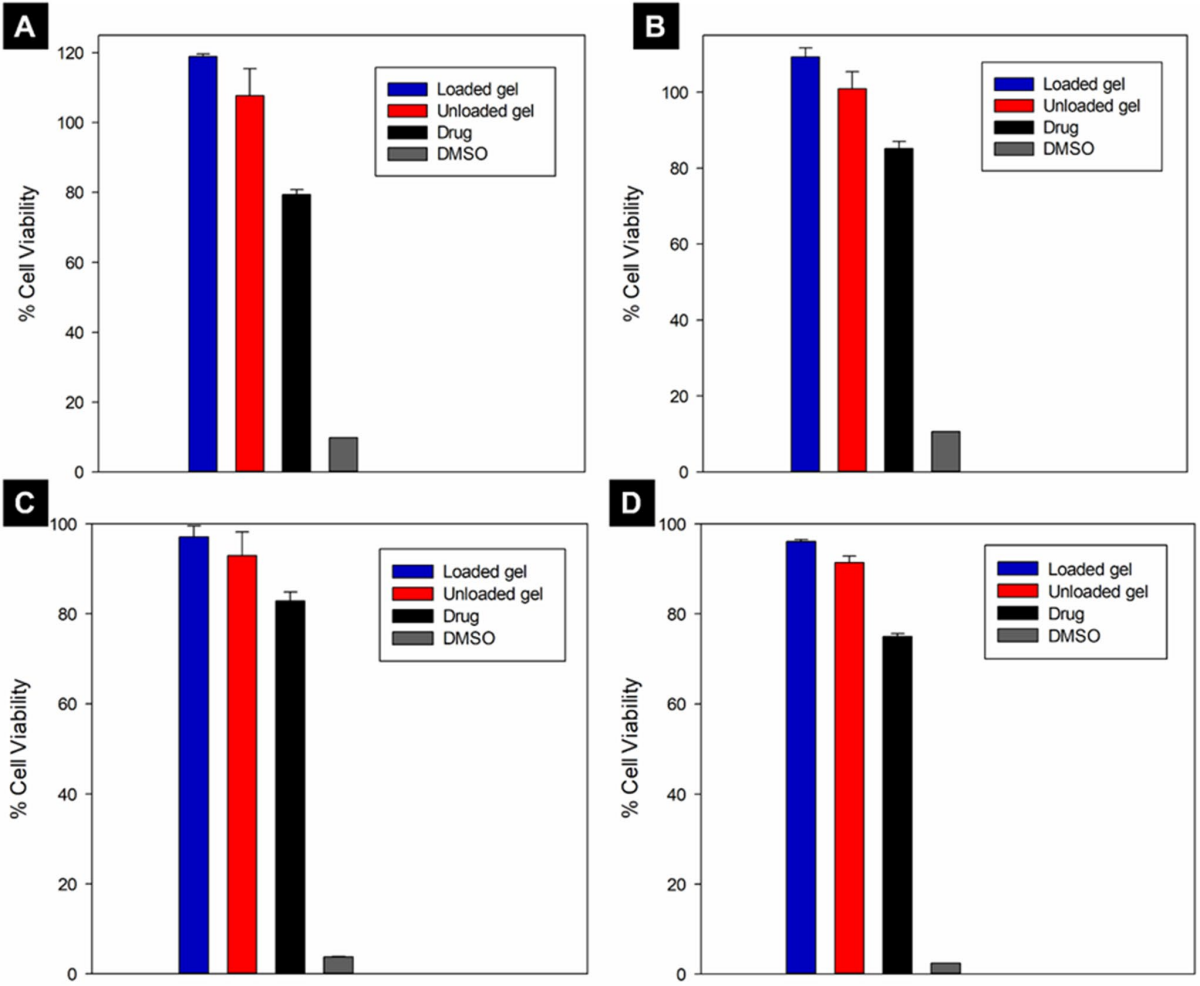
Cell biocompatibility with (**A**) 3T3 cells after 48 h, (**B**) 3T3 cells after 72 h, (**C**) hRPE cells after 48 h and, (**D**) hRPE cells after 72 h. All data are represented as mean ± S.D., *n* = 3

Biocompatibility studies were conducted on 3T3 and hRPE cell lines. Both cell lines were utilised in line with previous studies of potential vitreous substitutes [9, 22, 128, 129]. Studies with the hRPE cell line are particularly important as clinical administration of the hydrogels in the vitreous body would result in direct contact between the substitute and retinal pigment epithelium. The MTT cell viability assays (Fig. 9) showed that the unloaded and loaded hydrogels were not toxic to either cell line over 48- and 72 h and demonstrated enhanced biocompatibility compared to the pure drug (*p* < 0.05 for the loaded gel vs. pure drug; *p* > 0.05 the unloaded gel vs. pure drug in both cell lines). The cell viability of both cell lines was expressed as the relative value compared to the untreated control cells. The cell viability of the unloaded and loaded gel systems exceeded 100% in certain instances, as has been reported by other investigators for artificial vitreous substitutes containing hyaluronic acid [63].

### Pilot evaluation of in vivo drug release and biocompatibility in a rabbit model

#### Animal post-operative survival, recovery, and behaviour

All the animals maintained general well-being following the intravitreal administration of the loaded hydrogel. Rabbits were awake and responsive within an hour post-surgery and the lights of the room were left off to allow for acclimatisation of their treated eye. The rabbits were checked routinely, and no signs of infection were observed. Partial tightening of the orbit of the treated eye was observed in some rabbits on the first-day post-surgery (via comparison with the Rabbit Grimace Scale), reducing thereafter within the first week and not present in the last 3 weeks of the study. This corresponds with the initial observed swelling behaviour of the loaded hydrogel and may further be due to light sensitivity and tissue sensitivity as a result of the intravitreal injection. As this observation did not extend after the first week, any elevations in IOP can also not be attributed to TA release. The rabbits did not lose weight throughout the study and no obvious markers of discomfort were observed. This substantiates that the intravitreal administration of the nanoparticle-loaded hydrogel is technically feasible in the New Zealand White rabbits, and it is tolerated by the animals.

Macroscopic changes in the treated eye at each timepoint (Supplementary Fig. 4) are easily compared to the normal untreated rabbit eye (Supplementary Figs. 5 and 6). The treated eye of the rabbits euthanised 5 days post-surgery displayed macroscopic signs of inflammation, retraction of the orbit, engorgement of the iris vessels, and small amounts of blood in the anterior chamber. These are likely due to trauma from the surgical procedure. Iris vessel engorgement was reduced in the treated eyes of the rabbits that were euthanised 7 days post-administration and no blood was evident in the anterior chamber of the eye. Inflammation was still macroscopically visible with a slight retraction of the orbit of the treated eye. Inflammation was not macroscopically visible in the rabbits euthanised on days 14, 21 and 28 indicating that the inflammation observed in the first two groups may have been due to the surgical procedure, and TA release from the hydrogel would have assisted in the decrease in inflammation. These results are comparable to those reported by Swindle-Reilly et al. [130] and Uesugi et al. [131] with no significant inflammation or gel opacity reported. One of the rabbits developed a cataract due to the perioperative touching of the lens while three rabbits developed a cataract possibly due to the hydrogel causing protein or fibre breakdown in the lens of those eyes [132].

#### Histomorphological analysis

**Fig. 10:**
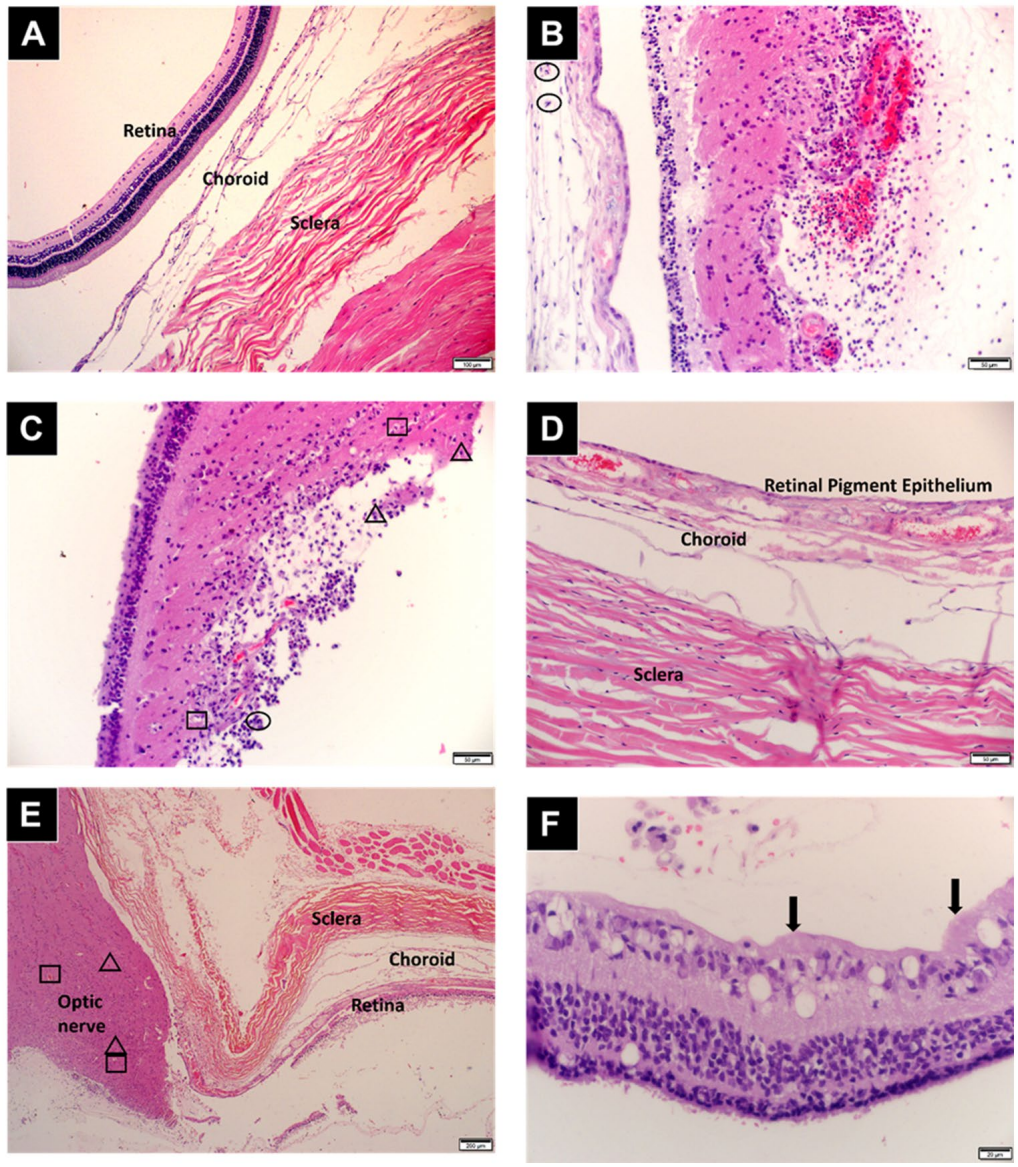
Histological slides of enucleated eyes at each time point. (**A**) Negative control eye showing the retina, choroid, and sclera at 10x magnification, B– F hydrogel administered eyes (Scale bar = 100 μm). (**B**) Day 5 shows the retina at 20x magnification (Scale bar = 50 μm), (**C**) Day 7 shows the retina at 20x magnification (Scale bar = 50 μm), (**D**) Day 14 shows the choroid and sclera at 20x magnification (Scale bar = 50 μm), (**E**) Day 21 shows the optic nerve, retina, choroid and sclera at 4x magnification (Scale bar = 200 μm), (**F**) Day 28 showing the retina at 40x magnification (Scale bar = 20 μm). Symbols in the histopathological images indicate the following: circle = heterophils; square = macrophages; triangle = lymphocytes; arrows = hypertrophy and tombstoning of epithelial cells. (All visualisations were conducted on 3 samples for each time point, *n* = 3. Representative images are shown.)

Haematoxylin and eosin (H&E) staining was conducted for the histomorphological analysis for assessing the degree of inflammation and the general histologic observation of the enucleated ocular tissues (Fig. 10). Morphological changes to the cornea were only observed in the enucleated eyes of the rabbits on day 5 suggesting that this was related to the surgical procedure. Mild inflammation (1+) was noted in the bulbar conjunctiva only in the eyes enucleated on day 5, likely due to the changes observed in the cornea. The sclera and periocular soft tissue of all the enucleated eyes were histologically normal and no signs of scleritis were observed. Changes to the choroid were observed for most of the eyes with mild to moderate choroiditis (1 + to 2+). These transformations are proposedly due to ischaemia and not necrosis as typical necrosis signs were not observed. The surface and superficial layers of the optic disc displayed inflammation due to its contact with the vitreous. Lens capsule protein fibres were visible in the vitreous body in some eyes. This may be of surgical origin or due to the piercing of the globe for aspiration of aqueous humour and vitreous humour for in vivo drug release studies. Lastly, the inner retinal surfaces that are in close proximity to the vitreous body displayed retinitis. Figure 10 depicts the changes in the retina over the 28 days of the study. The retina showed inflammation ranging from mild (1+) to moderate inflammation (2+) over the 28-day study period. This is observed with the presence of small numbers of heterophils, macrophages and lymphocytes in Fig. 10 (B,C & E). These inflammatory cell infiltrations observed in the retina were absent in the other ocular tissues one week following administration (with a mild presence of inflammatory material (1+) observed in the anterior chamber and periocular soft tissue of the rabbits only on Days 5 and 7).

A minority (5) of the enucleated eyes from day 7 to day 28 (two on day 7 and one each for days 14–28) displayed tombstoning of the outer retinal epithelial cells indicative of retinal detachment. The tombstoning or hypertrophy of the outer epithelia cells is displayed by the arrows in Fig. 10F. This observation is most likely attributed to the intravitreal injection procedure. Studies by Jakobsson et al. [133], Ben Yahia et al. [134], and Yasuda et al. [135] reported retinal detachment following intravitreal injections. Inflammation to the ocular tissues occurs post-intravitreal surgery in the majority of the studies and was expected [6, 132]. Histomorphological analysis of rabbit eyes following vitreous substitution conducted by Swindle-Reilly et al. [130] Uesugi et al. [131] and Santhanam et al. [136] demonstrated inflammatory cells localised to the site of vitrectomy and tissues in contact with the vitreous humour similar to those observed in this study [130, 131, 136]. These studies also reported no degenerative changes observed in the retina with H&E staining.

#### In vivo drug release

**Fig. 11:**
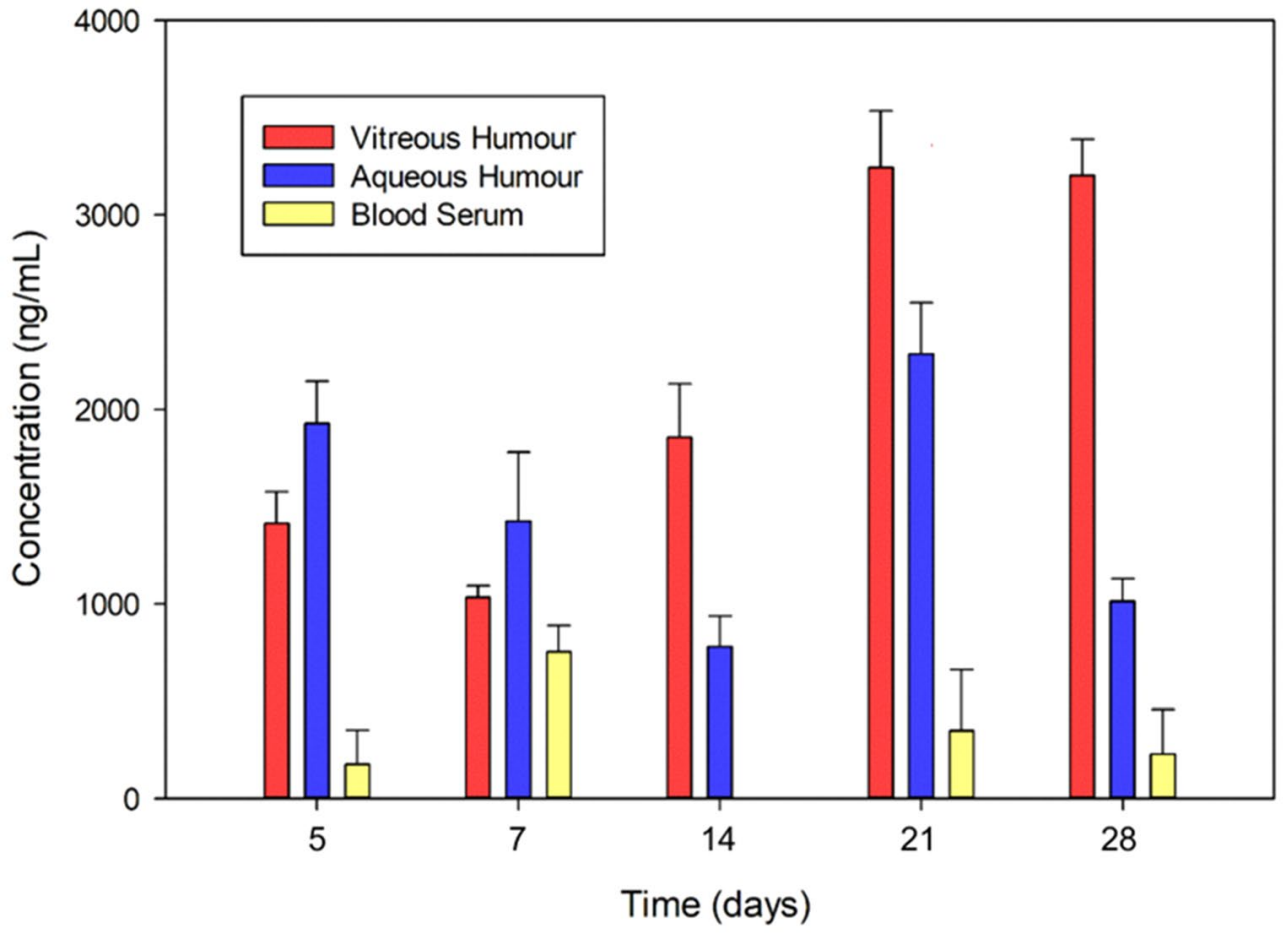
In vivo drug release in the vitreous humour, aqueous humour, and blood serum (mean ± S.D., *n* = 3)

A calibration curve for TA was constructed to analyse the UV-Vis absorbance readings obtained for the blood serum and aspirated vitreous humour and aqueous humour of the studied rabbits. The drug levels measured in the vitreous humour, expressed as the drug concentration at each time point, reflects the drug that had been released from the nanoparticles and was present within the vitreous humour. As highlighted in Section “Experimental design and surgical protocol” and “Euthanisation, enucleation and sampling”, measures were implemented to designate separate sites for injection of the vitreous substitute and withdrawal of vitreous humour samples for analysis in order to maximise sampling of the native vitreous rather than the substitute. The release of drug from these nanoparticles is retarded by the vitreous substitute initially, which thermoresponsively forms a gel depot within the surrounding vitreous humour on injection. Drug levels in the aqueous humour reflects free drug which has diffused from both the nanoparticles and vitreous substitute, and subsequently permeated across the blood-aqueous barrier into the anterior chamber. This highlights that although the substitute slows diffusion of the drug from the nanoparticles, the drug does diffuse from the actual substitute to avail therapeutically effective levels in the native aqueous and vitreous humour. Figure 11 depicts the concentration of drug measured in the blood serum, vitreous humour, and aqueous humour at all the euthanisation time points. Minimal amounts of drug were found in the blood indicative of localised drug release. Higher drug release in the blood serum on days 5 (174.91 ng/mL ± 233.211 ng/mL) and 7 (751.134 ng/mL ± 170.408 ng/mL) is potentially due to perioperative bleeding leading to drug release in the systemic circulation. TA concentrations within the vitreous and aqueous humour indicates localised drug release to ocular tissues with release detected for the duration of the study (28 days) (*p* > 0.05); and were significantly higher than blood serum levels (*p* < 0.05). Intravitreal TA has a minimum effective concentration (MEC) of 100 ng/mL [137] and a large therapeutic window [138]. Thus, the drug release levels achieved in vitreous and aqueous were maintained within the therapeutic window for the duration of the study and were within the range reported by other investigators for intravitreal TA administration at a similar dose of ~ 4–6 mg [139, 140]. However, compared to intravitreal injection, the vitreous substitute hydrogel developed herein demonstrated maintenance of TA levels within the therapeutic window over 28 days, indicative of the sustained release behaviour of the hydrogel system. This is advantageous as the intravitreal TA concentration has been reported to decrease over time following intravitreal injection of TA [139, 140]. The concentrations of TA following administration, specifically from day 14, were higher in the vitreous (1856.30 ng/mL ± 349.553 ng/mL) than the aqueous humour (780.03 ng/mL ± 185.750 ng/mL). This was also observed following TA administration by Kamppeter et al. [139]. and Zhang [140]. Drug levels increased in the vitreous humour, specifically, as more drug is released from the nanoparticles and as the substitute undergoes biodegradation within the vitreous chamber (posterior segment). Aqueous humour levels became comparatively less than vitreous levels due to lower drug diffusion into the anterior segment as the level of inflammation decreases, causing a decrease in the permeability of the blood-aqueous barrier.

In vitro drug concentrations at each time point were significantly higher than in vivo concentrations at corresponding time points (*p* < 0.05). Sustained release over an extended period is anticipated from the TA-loaded vitreous substitute in vivo, potentially due to interactions between the native vitreous and the HA-based substitute, further prolonging the release. There was a good correlation between the in vitro TA release profile and in vivo concentration profile of TA in the vitreous humour (R^2^ = 0.93), highlighting corresponding release behaviour in vitro and in vivo. However, specific *in vitro-in vivo* correlations would be drawn from a full preclinical assessment.

Spitzer et al. [44] investigated dexamethasone release in vitro from hyaluronic acid–based vitreous substitutes incorporating dexamethasone in concentrations reaching 20 mg/mL. The substitute provided diffusion-controlled release proceeded over 44 h and was not cytotoxic in the presence of human tenon fibroblasts and human retinal pigment epithelial cells (ARPE-19). In vivo release studies were not undertaken. Due to the diffusion-based kinetics and the use of uncrosslinked hyaluronic acid which underwent degradation in the vitreous cavity within a few weeks, this system would be limited to short-term vitreous replacement, whereas the system reported herein would provide a more prolonged vitreous replacement and anti-inflammatory delivery system.

## Conclusion

The present study assessed the potential of PLGA nanoparticles entrapped in a hyaluronic acid-poloxamer blend hydrogel as a vitreous substitute and drug delivery system via in-depth in vitro characterisations of the progressive hydrogel system developed, as well as pilot in vivo analysis to highlight its translation potential. In this investigation, unloaded and nanoparticle-loaded hydrogels were prepared that displayed similar optical and viscoelastic properties to the natural vitreous. Rheological studies showed the ability of the hydrogels to form in situ, and both hydrogels can be injected via similar gauge needles as those currently used clinically. Furthermore, the hydrogels demonstrated excellent swelling and degradation behaviour furthering their potential to be suited as vitreous substitutes. Additionally, the nanoparticle-loaded hydrogel showed long-term drug release of the model anti-inflammatory hydrophobic drug, TA, and thus has the potential for delivery of other hydrophobic ocular therapeutics for the treatment of posterior segment/ vitreoretinal diseases. Lastly, both hydrogels displayed in vitro biocompatibility with two cell lines including a retinal cell line.

The objective to inject the nanoparticle-loaded hydrogel into an NZW rabbit model was successfully achieved with no loss of life, no post-operative infection, no complete vision loss in the treated eye, and moderate inflammation. While the loaded hydrogel achieved success in many areas, further in vivo analyses are required for comprehensive assessment of the potential of the hydrogel to function as a long-term vitreous substitute; the initial pilot study is reported herein. In vivo drug release analysis showed the localised release of TA at therapeutic levels in the vitreous and aqueous humour throughout the period of investigation (28 days) indicating that the hydrogel remained in the vitreous. Histomorphological analysis showed higher inflammation in the surrounding ocular tissues in the first week, reducing thereafter for the remaining 3 weeks. This may be due to the initial inflammation as a result of the surgical procedure, followed by the anti-inflammatory effect with the release of the corticosteroid drug over the 28 days, thus reducing intraocular inflammation.

These results suggest that PLGA nanoparticles loaded within a HA-Poloxamer in situ-forming hydrogel is a promising candidate for vitreous substitution with sustained localised drug delivery for the enhanced precision treatment of various posterior segment diseases.

## Electronic supplementary material

Below is the link to the electronic supplementary material.

**Supplementary Figure 1** - DSC thermograms of all polymers, triamcinolone acetonide and all nanoparticle and hydrogel formulations.

**Supplementary Figure 2** - TGA thermograms of the unloaded and loaded nanoparticles, and the unloaded and loaded hydrogels.

**Supplementary Figure 3** - X-Ray Diffractograms of the polymers, drug and nanoparticle and hydrogel formulations.

**Supplementary Figure 4** - Macroscopic view of the treated rabbit eye A 1-3) Day 5, B 1-3) Day 7, C 1-3) Day 14, D 1-3) Day 21 and E 1-3) Day 28.

**Supplementary Figure 5** - Macroscopic view of the untreated rabbit eye.

## Data Availability

All data generated or analysed during the current study are available from the corresponding author on reasonable request.
