# Supplementary figures and images for: In vivo evaluation of a Nano-enabled therapeutic vitreous substitute for the precise delivery of triamcinolone to the posterior segment of the eye
Source: Drug Deliv Transl Res. 2024 Mar 22;14(10):2668–94. doi: 10.1007/s13346-024-01566-1 (PMC11384602; doi:10.1007/s13346-024-01566-1)

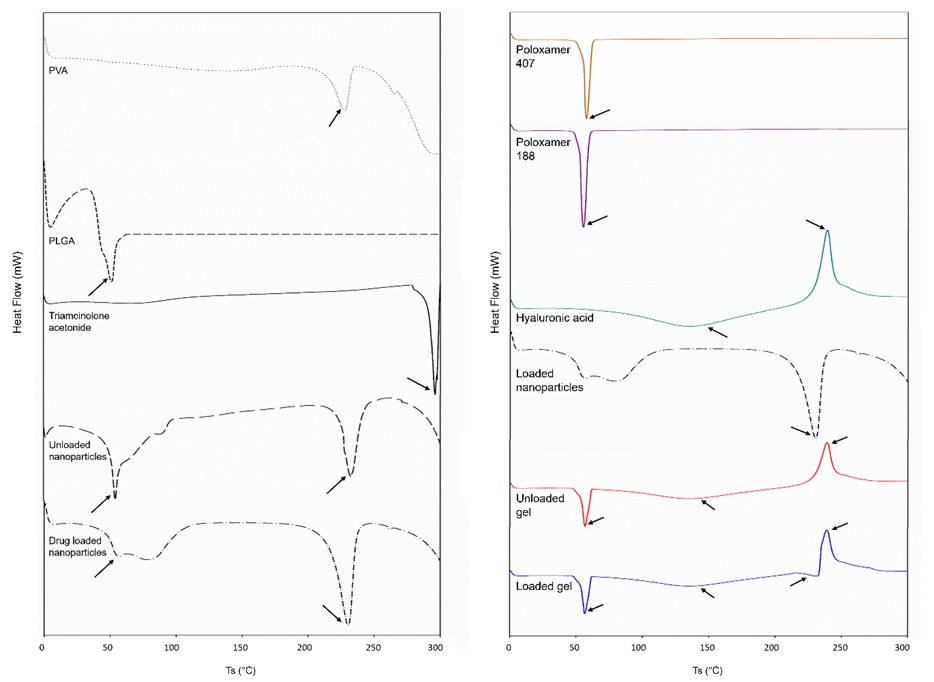

Supplement: Supplementary file 1 — Supplementary Figure 1 - DSC thermograms of all polymers, triamcinolone acetonide and all nanoparticle and hydrogel formulations. [file 13346_2024_1566_MOESM2_ESM.tiff]

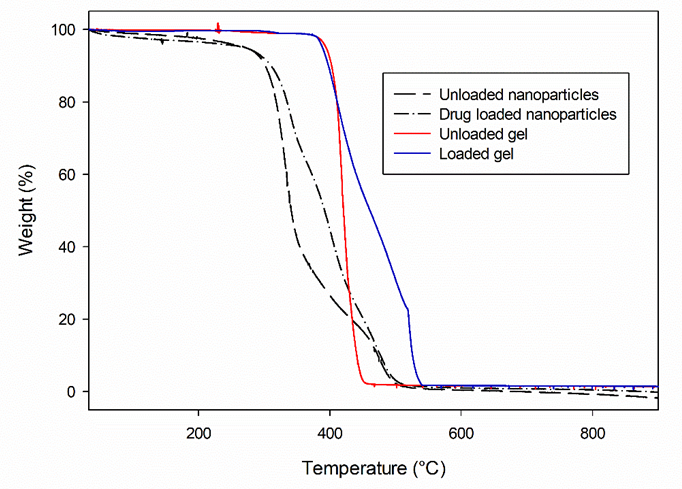

Supplement: Supplementary file 2 — Supplementary Figure 2 - TGA thermograms of the unloaded and loaded nanoparticles, and the unloaded and loaded hydrogels. [file 13346_2024_1566_MOESM3_ESM.tiff]

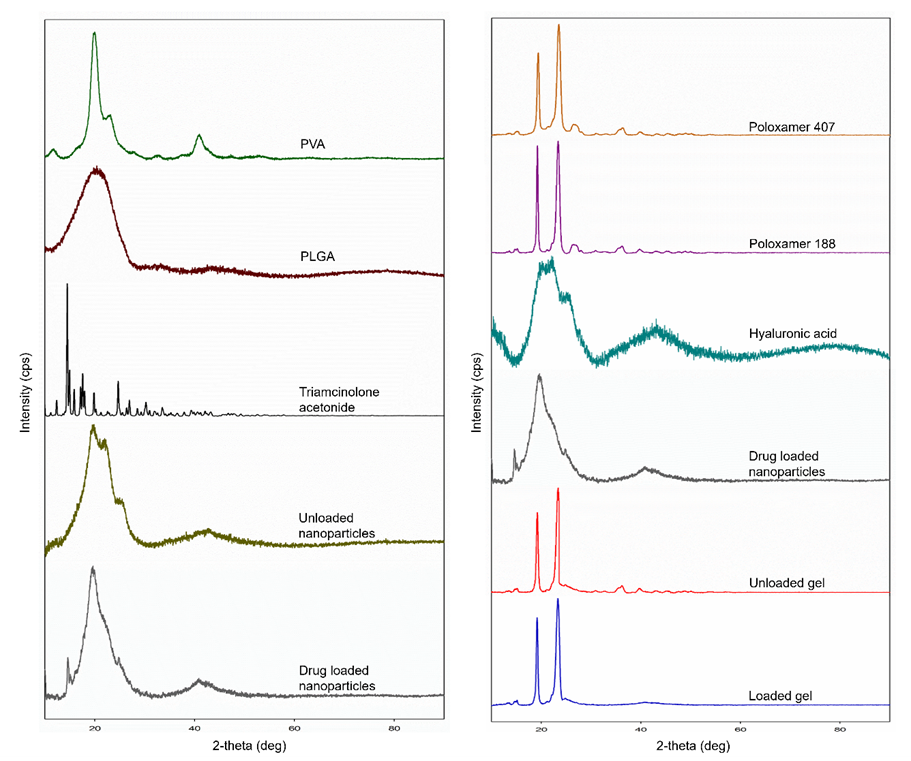

Supplement: Supplementary file 3 — Supplementary Figure 3 - X-Ray Diffractograms of the polymers, drug and nanoparticle and hydrogel formulations. [file 13346_2024_1566_MOESM4_ESM.tiff]

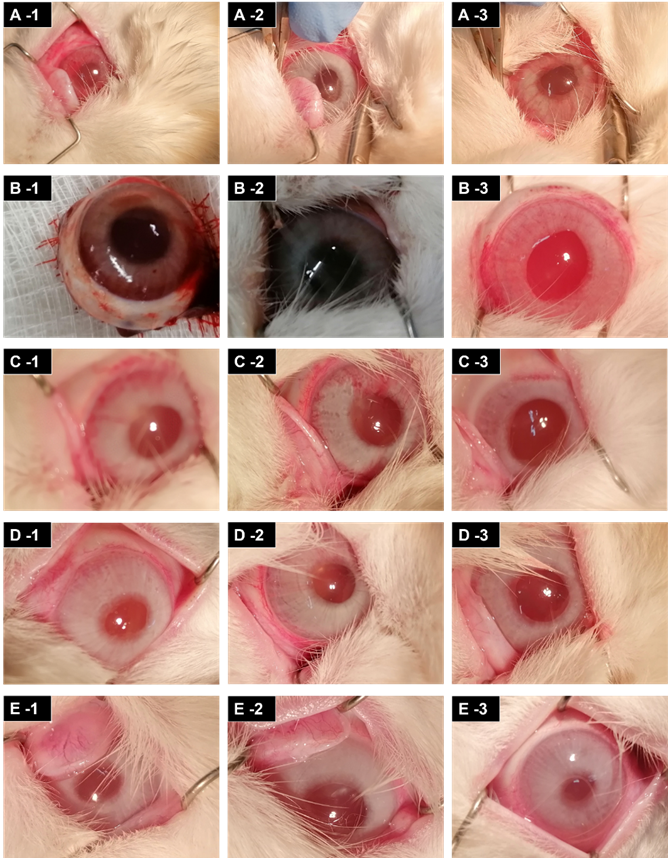

Supplement: Supplementary file 4 — Supplementary Figure 4 - Macroscopic view of the treated rabbit eye A 1-3) Day 5, B 1-3) Day 7, C 1-3) Day 14, D 1-3) Day 21 and E 1-3) Day 28. [file 13346_2024_1566_MOESM5_ESM.tiff]

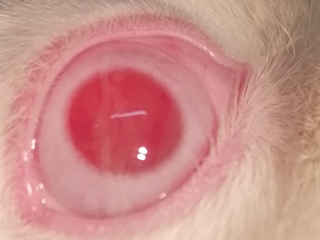

Supplement: Supplementary file 5 — Supplementary Figure 5 - Macroscopic view of the untreated rabbit eye. [file 13346_2024_1566_MOESM6_ESM.tiff]
